# Immuno-activated mesenchymal stem cell living electrospun nanofibers for promoting diabetic wound repair

**DOI:** 10.1186/s12951-022-01503-9

**Published:** 2022-06-21

**Authors:** Shaoying Gao, Tao Chen, Zhen Wang, Ping Ji, Lin Xu, Wenguo Cui, Ying Wang

**Affiliations:** 1 Department of Burn and Plastic surgery, Affiliated Hospital of Zunyi Medical University, Zunyi, 563000 Guizhou China; 2 Shanghai Institute of Immunology, Department of Immunology and Microbiology, Key Laboratory of Cell Differentiation and Apoptosis of Chinese Ministry of Education, Shanghai Jiao Tong University School of Medicine, Shanghai, 200025 China; 3 Shanghai Key Laboratory for Prevention and Treatment of Bone and Joint Diseases, Shanghai Institute of Traumatology and Orthopaedics, Ruijin Hospital, Shanghai Jiao Tong University School of Medicine, 197 Ruijin Second Road, Shanghai, 200025 People’s Republic of China; 4 Department of Immunology, Special Key Laboratory of Gene Detection and Therapy & Base for Talents in Biotherapy of Guizhou Province, Zunyi, 563000 China

**Keywords:** Diabetic wound healing, Macrophage cell membrane, Electrospun nanofibers, Bone marrow-derived mesenchymal stem cells

## Abstract

Diabetic wound is the leading cause of non-traumatic amputations in which oxidative stress and chronic inflammation are main factors affecting wound healing. Although mesenchymal stem cells (MSCs) as living materials can promote skin regeneration, they are still vulnerable to oxidative stress which limits their clinical applications. Herein, we have prepared (polylactic-co-glycolic acid) (PLGA) nanofibers electrospun with LPS/IFN-γ activated macrophage cell membrane. After defining physicochemical properties of the nanofibers modified by LPS/IFN-γ activated mouse RAW264.7 cell derived membrane (RCM-fibers), we demonstrated that the RCM-fibers improved BMMSC proliferation and keratinocyte migration upon oxidative stress in vitro. Moreover, bone marrow derived MSCs (BMMSCs)-loaded RCM-fibers (RCM-fiber-BMMSCs) accelerated wound closure accompanied by rapid re-epithelialization, collagen remodeling, antioxidant stress and angiogenesis in experimental diabetic wound healing in vivo. Transcriptome analysis revealed the upregulation of genes related to wound healing in BMMSCs when co-cultured with the RCM-fibers. Enhanced healing capacity of RCM-fiber-BMMSCs living material was partially mediated through CD200-CD200R interaction. Similarly, LPS/IFN-γ activated THP-1 cell membrane coated nanofibers (TCM-fibers) exhibited similar improvement of human BMMSCs (hBMMSCs) on diabetic wound healing in vivo. Our results thus demonstrate that LPS/IFN-γ activated macrophage cell membrane-modified nanofibers can in situ immunostimulate the biofunctions of BMMSCs, making this novel living material promising in wound repair of human diabetes.

**Graphical Abstract:**

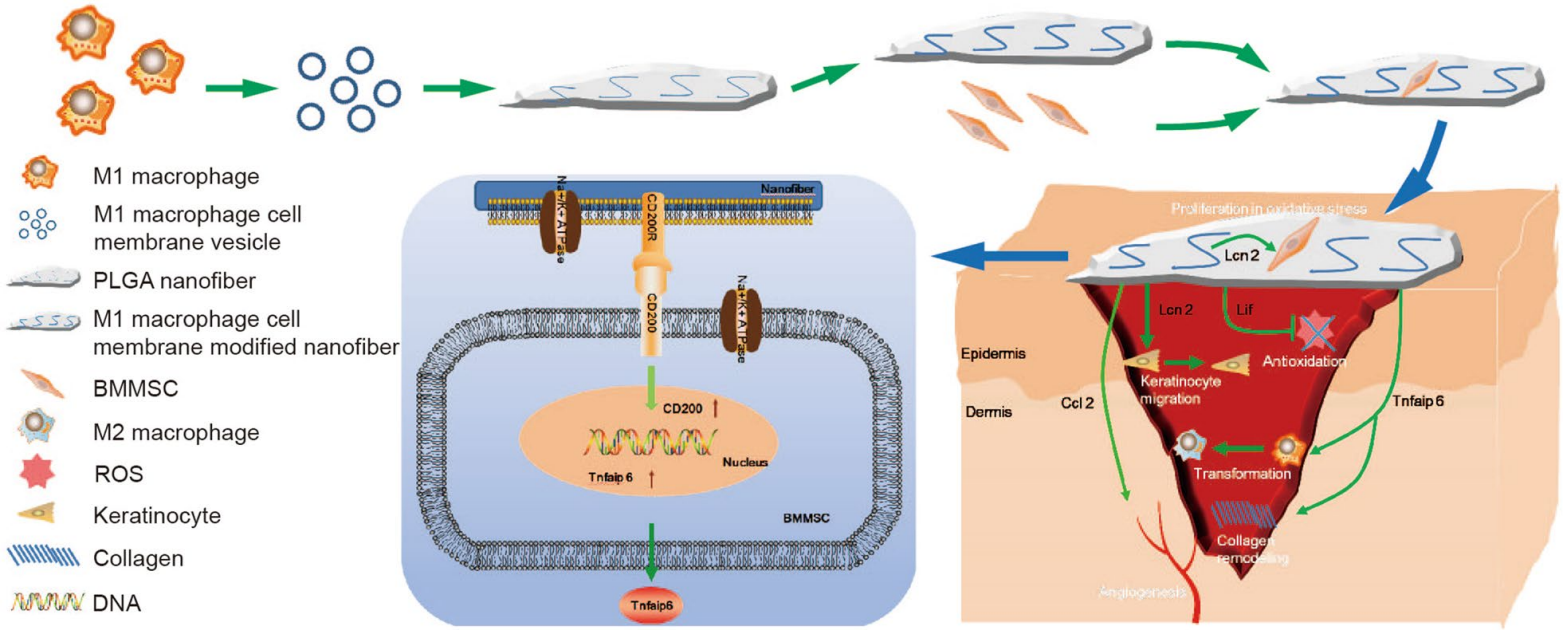

**Supplementary Information:**

The online version contains supplementary material available at 10.1186/s12951-022-01503-9.

## Introduction

Conventional biomaterials like nanofibers or hydrogels provide biophysical support as well as integrate bioactive molecules to instruct biological functionality in tissue regeneration [1], making them successful in plenty of pre-clinical investigations. Considering our tissues are composed of living cells and cell-secreted extracellular matrices (ECMs) capable of adapting to diverse biological scenarios, living materials have been conceptualized and further engineered. These engineered living materials are capable of generating biologics responding to local environment and yielding more adaptations through autonomously biological behaviors [2]. At present, bacteria, fungi and stem cells have been used in living materials for a range of applications such as biosensing, tissue regeneration and drug delivery etc. [3–6]. Living materials exhibit “living characters” through loading living cells on polymeric matrice blocks to construct bioactive and bioresponsive units [7]. Until now, living materials containing mesenchymal stem cells (MSC), keratinocytes or fibroblasts have been mainly recommended in wound care and wound management. These living cells are demonstrated to be able to ameliorate wound healing processes or belong to main components in the wound regions to be reprogrammed during wound healing [8]. Polymeric matrices-based living materials are therefore more likely to improve regenerative performances over conventional biomaterials partially through providing more cell adhesion capability, maintaining the merits and characteristics of living cells such as MSCs, as well as promoting cell survival, proliferation, and differentiation by mediating cell-matrix interactions within in vivo microenvironment [9].

Diabetes associated chronic wounds have become one of the main common complications among non-traumatic amputations nowadays. Although diabetic wound care is managed routinely such as haemostasis, anti-inflammation, cell proliferation and tissue remodeling [10, 11], it is still refractory to currently available treatment for chronic wounds owing to several unsolved difficulties including increased regional oxidative stress, dysfunctionality of immune cells and keratinocytes as well as impaired angiogenesis *etc*. [12]. In addition, high glucose and subsequent oxidative stress in wound regions of diabetic patients also cause severe damages to the cells when using living cell-containing biomaterials. Considering the alterations of internal environment suffering from chronic diseases like diabetes, living cell-containing biomaterials might be affected to execute normal function. The design of living materials according to pathologic circumstance for wound healing still needs inspiration.

Nanofiber living materials not only provide the scaffolds for cell attachment and proliferation, but also supply bioactive cues to resident cells [13]. The interactions between the cells and the nanofibers are of particular importance to determine the survival and function of resident cells [14]. Therefore, nanofibers have been modified with cell surface receptors for intracellular signaling, which alters protein expression and modulate the functions of resident cells [15]. Although these bottom-up functionalization approaches are sufficient to present individual receptor or receptor combinations to resident cells in the scaffolds, they are generally inadequate for recapitulating the complexity and functions during cell-cell interactions [16]. Therefore, cell membranes derived from living cells are superior to provide *bona fide* cell surface receptors with integrity and functionality [17]. Polymeric nanofibers coated directly with cell membranes thus become a new and robust strategy to improve the bioactivity of living materials. In fact, the use of cell membranes to modify synthetic biomaterials through a top-down fabrication method has emerged firstly as a promising technique for nanomaterial modification [18]. A variety of cell membrane-coated nanoparticle systems have been developed with unique features and functions to support resident cells. Cell membranes from different cell types (for instance red blood cells, platelets, leukocytes, cancer cells or bacteria) are incorporated within synthetic nanoparticles such as polymeric nanoparticles, gold nanoparticles, and silica nanoparticles [19]. These nanoparticles have been used in a wide range of biomedical applications including drug delivery, photodynamic therapy, detoxification, and vaccination [20].

It has been addressed that interferon (IFN)-γ activated MSCs exhibited stronger wound healing functions than resting MSCs [21]. In addition, plenty of studies have demonstrated that the interactions between macrophages and MSCs supported biological functions and behaviors of MSCs both in vitro and in vivo [22–24]. Considering that macrophages exert the modulations on bone marrow derived MSCs (BMMSCs) through cell-cell contact [25], whether macrophage cell membrane coated nanofiber scaffolds have in situ immunostimulation on BMMSCs with living material properties is worthy of exploration. The efficacy of BMMSCs-loading nanofiber living materials on improving wound healing under pathologic diabetes circumstance also needs to be clarified.

**Scheme 1:**
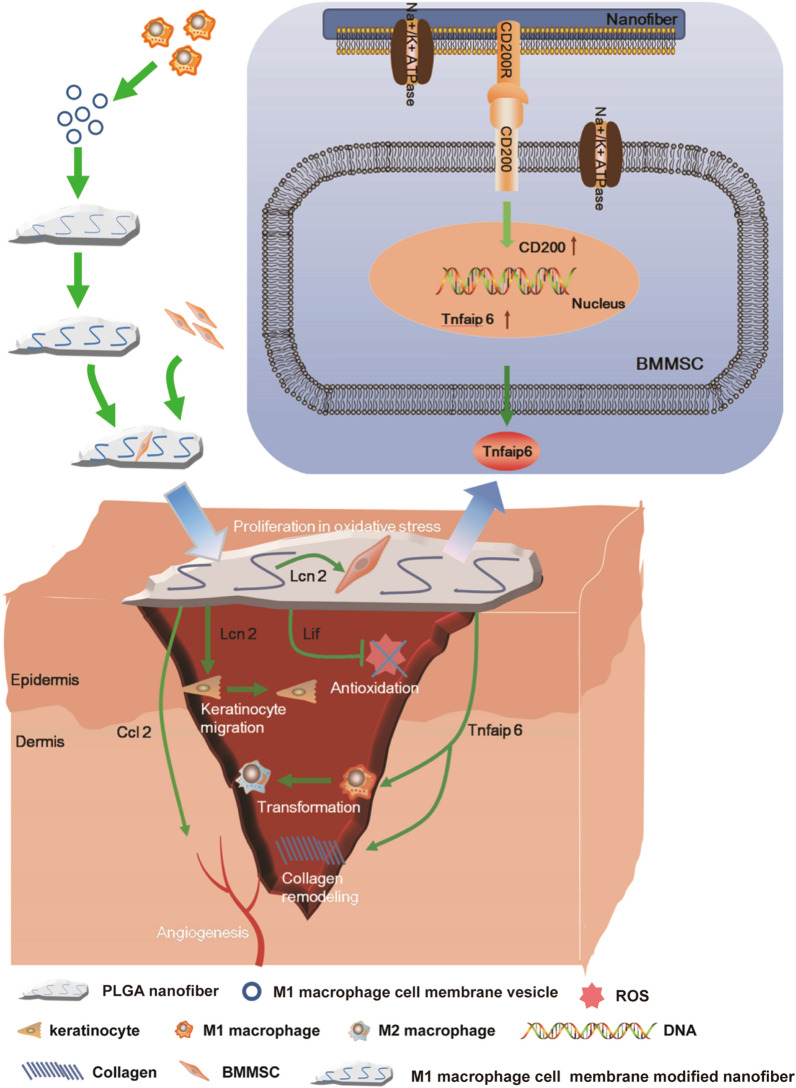
LPS/IFN-γ activated RAW264.7 cell (M1-like murine macrophage cell) membrane modified PLGA nanofibers with BMMSCs attachment promote diabetic wound healing. The RCM-fiber-BMMSCs accelerate wound closure with rapid re-epithelialization, collagen remodeling, immunoregulation, antioxidant stress and angiogenesis in experimental diabetic wound healing in vivo. This is partially achieved through CD200-CD200R axis accompanied by the upregulation of *Lcn2, TNFAIP6, Lif* and *Ccl2* in BMMSCs. The RCM-fiber-BMMSCs function as a living material with in situ immunostimulatory capacity to exaggerate the biofunctions of BMMSCs

In this study, polylactic-co-glycolic acid (PLGA) nanofibers, a scaffold material being approved for several biomedical applications including tissue engineering [26–28], were electrospun with the cytomembrane from Lipopolysaccharide (LPS)/IFN-γ activated macrophages and further used to load BMMSCs to construct a novel living material. We firstly addressed biophysical properties of the nanofibers modified by LPS/IFN-γ activated mouse RAW264.7 cell membrane (RCM-fibers) including the diameter, hydrophilicity and degradation rate. We further demonstrated that the RCM-fibers could promote the proliferation of BMMSCs and keratinocyte migration under oxidative stress in vitro. When constructing RCM-fiber-BMMSCs as a living material, we found that RCM-fiber-BMMSCs accelerated diabetic wound healing with rapid re-epithelialization, collagen remodeling, more resistance to antioxidant stress and better angiogenesis when compared to Un-fiber-BMMSCs (nanofibers carrying BMMSCs without cell membrane modification). Based on transcriptome analysis of BMMSCs after co-culturing with the RCM-fibers, it was found that improved healing functions of RCM-fiber-BMMSCs were partially realized through CD200-CD200R interactions (Scheme 1). Of note, we also demonstrated that the nanofibers modified by the cytomembrane from LPS/IFN-γ activated human THP-1 cells (TCM-fibers) exhibited similar effects on promoting human BMMSC (hBMMSC) proliferation and keratinocyte migration upon oxidative stress. Being a living material, TCM-fiber-hBMMSCs also exhibited the advantages in diabetic wound healing. These results support that LPS/IFN-γ activated macrophage cell membrane-modified nanofibers can in situ immuno-stimulate BMMSCs, making this novel living material promising in diabetic wound repair.

## Results

### RCM-fibers promote the proliferation of mouse BMMSCs and enhance their resistance to oxidative stress

**Fig. 1:**
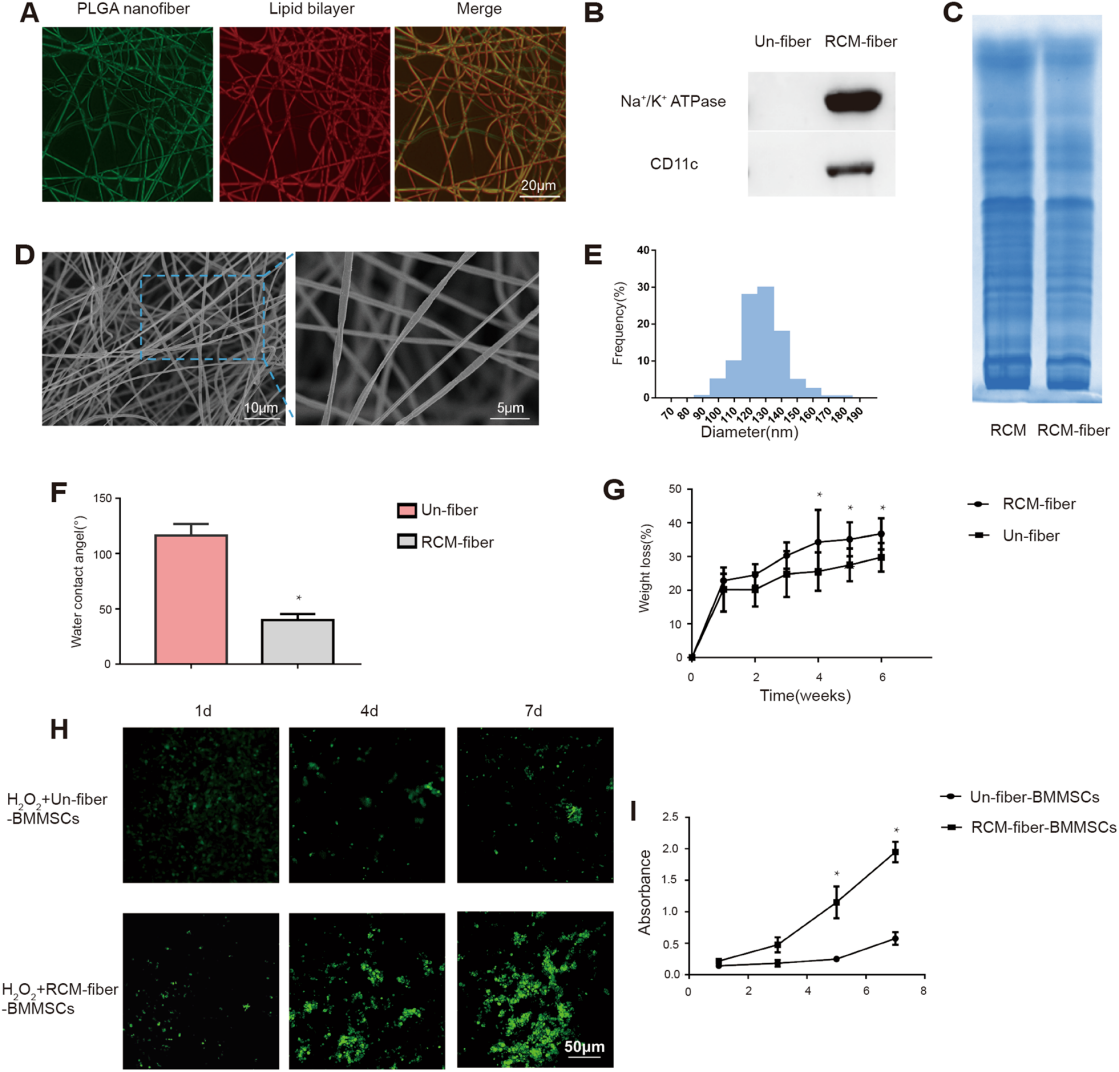
Physiochemical and biological characterization of the RCM-fibers.** A** Representative fluorescent images of the RCM-fibers after coumarin (for PLGA nanofiber) and Dil (for lipid bilayer) staining. Scale bar = 20 μm. **B** Detection of Na^+^/K^+^ ATPase and CD11c proteins on the RCM-fibers (with LPS/IFN-γ activated RAW264.7 cell membrane) and Un-fibers (without cell membrane) by Western blotting. **C** SDS-PAGE analysis of protein profiles of LPS/IFN- γ activated RAW264.7 cell membrane (RCM) and the RCM-fibers. **D** Representative images of the RCM-fibers morphology by the scanning electron microscopy. Scale bar = 10 μm (left) or 5 μm (right). **E** Size distribution of the RCM-fibers. **F** Comparisons of water contact angels between the Un-fibers and the RCM-fibers. **G** Comparisons of degradation rates between the Un-fibers and the RCM-fibers. **H** Fluorescent images of BMMSCs cultured on the Un-fibers or the RCM-fibers by CFDA-SE staining. Scale bar = 50 μm. **I** CCK-8 assays on cell viability of BMMSCs at day 1, day 3, day 5 and day 7 with the presence of H_2_O_2_. Data were represented as mean ± SD. Differences were assessed by using one-way ANOVA with Tukey’s multiple comparison tests. *P < 0.05, **P < 0.01

Although extensive studies have demonstrated that BMMSCs were able to accelerate cutaneous wound healing [29], their vulnerability to environmental stress limits their long-term efficacy in clinic. We therefore prepared cell membrane from mouse macrophage cell line RAW264.7 that was pre-treated with LPS and IFN-γ in vitro and electrospun on PLGA nanofiber scaffolds to prepare the RCM-fibers. The distribution of RAW264.7 cell membrane on the nanofibers was confirmed by coumarin (green) and Dil (red) staining, two lipophilic reporters for cell membrane (Fig. 1A). Western blotting analysis further demonstrated the enrichment of Na^+^/K^+^-ATPase (a marker specific for plasma membrane ) and CD11c (a marker specific for macrophage cell membrane) on the RCM-fibers whereas no Na^+^/K^+^-ATPase and CD11c were detected on the Un-fibers (the nanofibers without cell membrane) (Fig. 1B). Based on Coomassie blue staining protein profiles from the RCM-fibers were similar to those from cell membrane of LPS/IFN-γ activated RAW264.7 (Fig. 1C). By using a scanning electron microscope (SEM), we found that the RCM-fibers had a smooth outer surface and long fibrous morphology (Fig. 1D). The nanofibers loaded with cell membrane exhibited the diameters mainly in the range between 100–150 nm (Fig. 1E), which is aligned with the previous report [30]. In addition, the RCM-fibers exhibited significantly improved hydrophilicity with lower water contact angel when compared to the Un-fibers (Fig. 1F). The degradation rates of the Un-fibers were slower than those of the RCM-fibers (Fig. 1G).

Next we cultured BMMSCs with either the RCM-fibers or the Un-fibers exposed to H_2_O_2_ for 1, 4 and 7 days, and determined the proliferation of BMMSCs through CFDA-SE staining for living cells. On day 1, no obvious difference in green fluorescence was observed between two types of the nanofibers. However, on day 4 and day 7, more green fluorescence with the RCM-fibers was detectable when compared to that with the Un-fibers, demonstrating better proliferation of BMMSCs on the RCM-fibers (Fig. 1H). We also used the CCK-8 assay to determine the proliferation of BMMSCs with either the RCM-fibers or the Un-fibers upon oxidative stress induced by H_2_O_2_. It was similar that the RCM-fibers supported the proliferation of BMMSCs with more extent when compared to the Un-fibers (*P* < 0.05) (Fig. 1I). Therefore, PLGA electrospun nanofibers loading LPS/IFN-γ-treated RAW264.7 cell membrane (the RCM-fibers) are more favorable to support the proliferation of BMMSCs than the Un-fibers under oxidative stress in vitro.

### RCM-fiber-BMMSCs accelerate wound closure accompanied by collagen remodeling and increased neovascularization in the wound areas of experimental diabetic mice

**Fig. 2:**
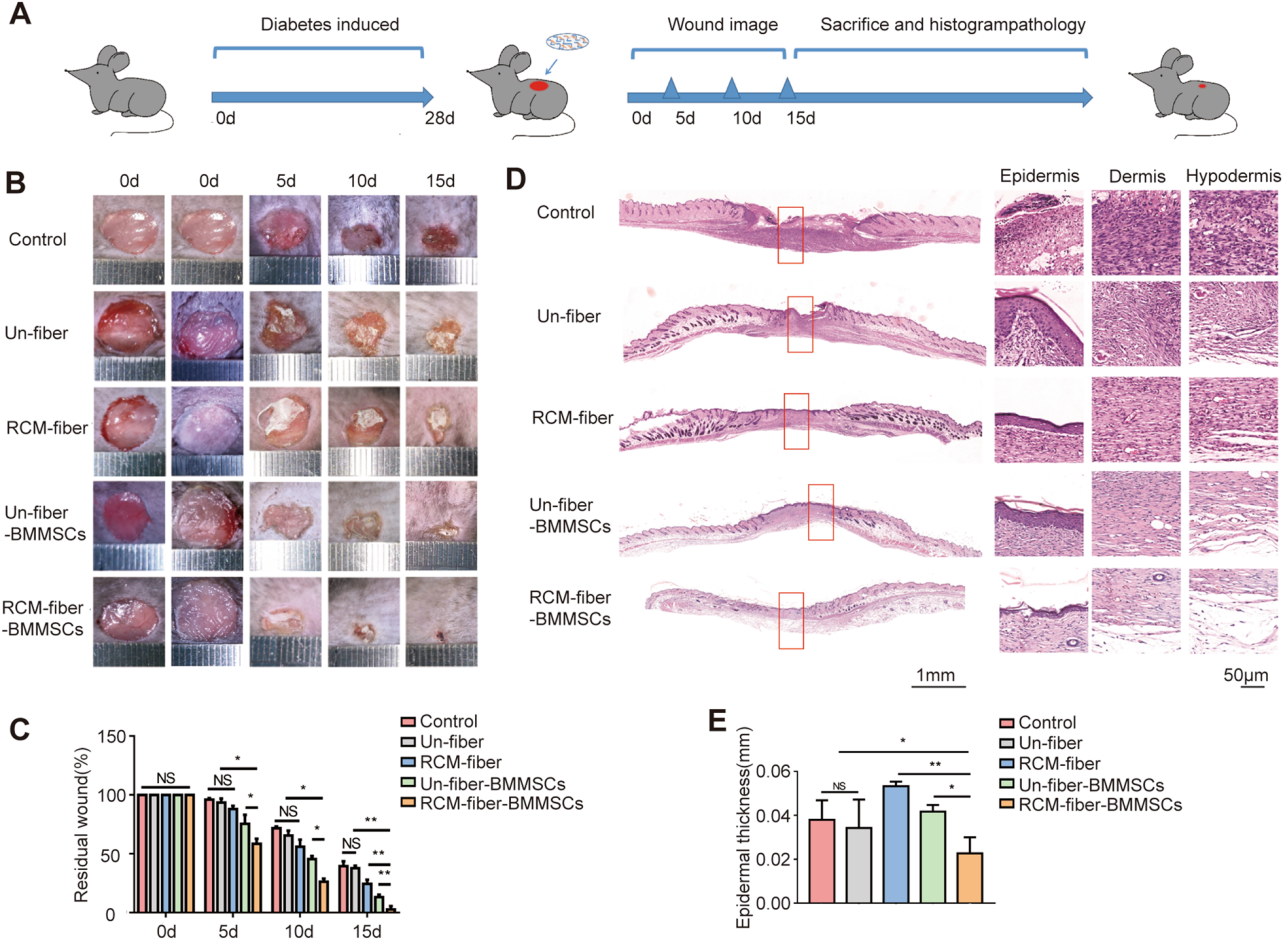
RCM-fiber-BMMSCs accelerate wound closure in diabetic mouse. **A **Experiment designing for diabetes induction followed by wound regeneration and healing processes. Mice were fed with high-fat and high sugar diets, and diabetes was induced by intraperitoneal injection of streptozocin. The wounds were made after 4 weeks of diabetes induction, and wound closure was studied in 2 weeks upon the treatment. **B** Representative images of the wounds at day 0, 5, 10 and 15 post-wounding, respectively. **C** Quantification of the residual wound areas at day 0, 5, 10 and 15, respectively. **D** Representative images of H&E staining for the wounds at day 15 (left) with high-resolution images of epidermis, dermis and hypodermis (right). Scale bar = 1 mm (left) or 50 μm (right). **E** Quantification of average epidermal thickness of the wounds. Data were represented as mean ± SD. Differences were assessed by using two-way analysis of variance (ANOVA) with Tukey’s multiple comparison tests and one-way ANOVA with Tukey’s multiple comparison tests. (mice: n ≥ 8) *NS*: non-significant, *P < 0.05, **P < 0.01

Since the RCM-fibers possessed more advantages in promoting the proliferation of BMMSCs in vitro, we next seeded mouse BMMSCs on the RCM-fibers to prepare the RCM-fiber-BMMSCs as a living material and investigated their effects on wound repair in experimental diabetes mice (Fig. 2A). No difference in body weight and blood glucose levels was observed among mice receiving different treatments (Additional file 1: Fig. S1). It was apparent that there displayed dramatic increase in wound closure rates in the RCM-fiber-BMMSCs treated group when compared to other four groups from day 5 (Fig. 2B). When calculating the remaining wound areas in different groups at day 15, we found that mice from RCM-fiber-BMMSCs treated group showed the smallest wound area (2.94 ± 2.26% to day 0) when compared to those from Un-fiber-BMMSCs (13.09 ± 1.90%), RCM-fibers (24.05 ± 2.81%) or Un-fibers (37.28 ± 1.71%) treated groups as well as control groups (38.58 ± 3.03%) with significant difference (Fig. 2C).

Wound re-epithelialization is one of the key features to evaluate the outcome of wound healing which can be evaluated by epidermal thickness. During skin regeneration, keratinocytes are inclined to activate leading to epidermal thickening and subsequent differentiation. We would speculate that RCM-fiber and Un-fiber-BMMSCs treated wounds experience incomplete differentiation of keratinocytes and undergo further differentiation and remodeling. We found that epidermal thickness in the wound regions of RCM-fiber-BMMSCs treated group at day 15 (the middle part in the panorama picture) was mostly equivalent to that of normal epidermis (the left and right sides of the wound regions in the panorama picture) (Fig. 2D). Epidermal thickness in the wound regions from RCM-fiber-BMMSCs treated group was 0.02 ± 0.006 mm while the average thickness of normal skins adjacent to the wound regions were 0.01 ± 0.004 mm. However, there displayed more epidermal thickness of the wound regions in other four groups when compared to those from RCM-fiber-BMMSCs treated group or normal skin (Fig. 2E). Moreover, the regenerated skins in RCM-fiber-BMMSCs treated group have developed mature epithelial structures such as hair follicles with less thin skin (Fig. 2D), further demonstrating rapid regeneration of epithelial skins when treated with the RCM-fiber-BMMSCs.

**Fig. 3:**
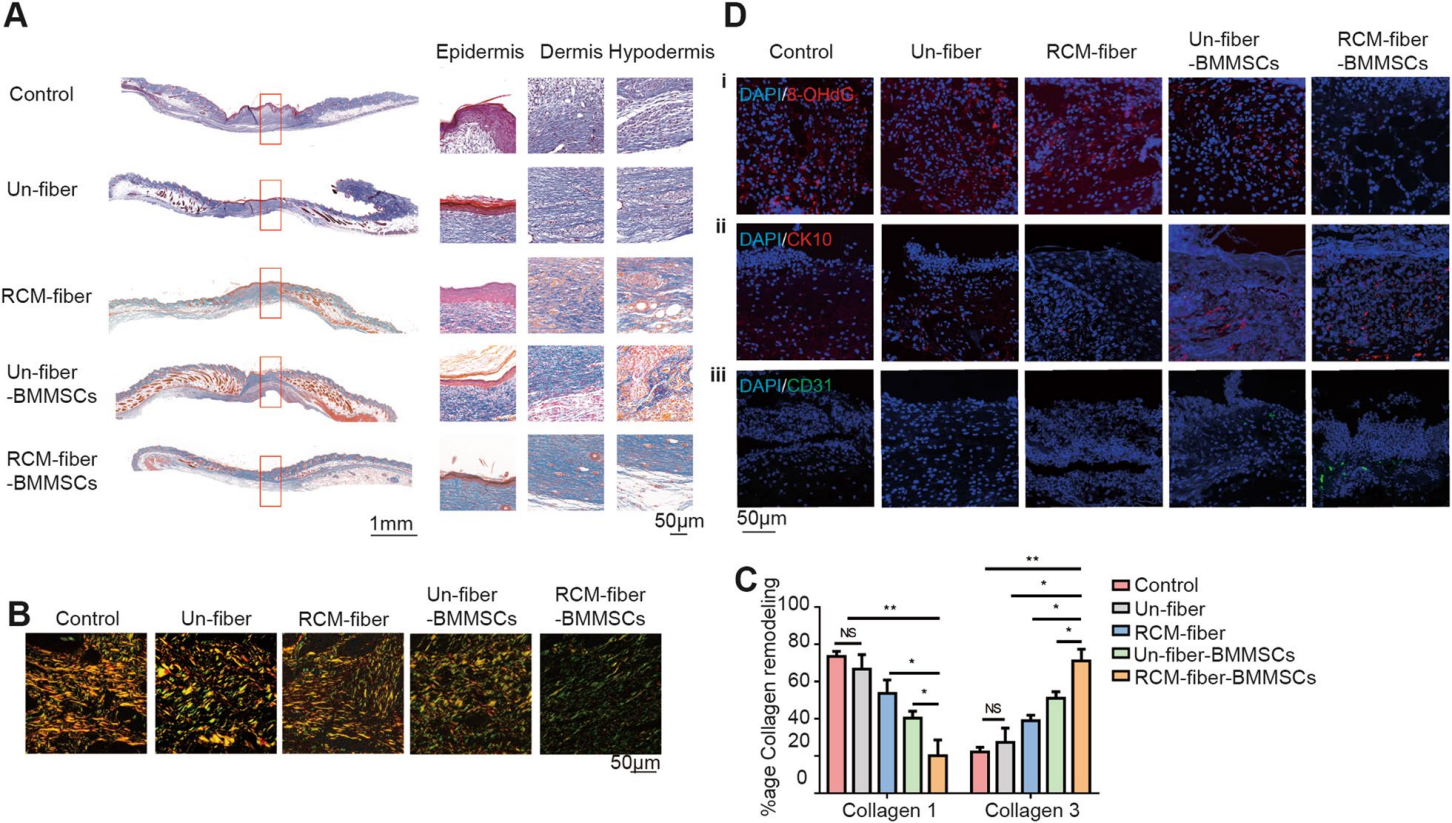
RCM-fiber-BMMSCs remodel collagen deposition at the wound areas.** A** Masson’s trichrome staining for tissue sections (left) with high-resolution images of collagen deposition in the epidermis, dermis and hypodermis (right) in diabetic mouse wounds. Scale bar = 1 mm (left) or 50 μm (right). **B** Picrosirius red staining for the collagens in the wound regions of diabetic mice after the treatment at day 15. Scale bar = 50 μm. **C** Comparisons of Collagen 1 and Collagen 3 percentages in the wound areas among five groups. **D** Immunostaining for 8-OHdG (i), CK10 (ii) and CD31 (iii) in the wound areas upon different treatments. Scale bar = 50 μm. Data were represented as mean ± SD. Differences were analyzed by one-way ANOVA followed by Tukey’s multiple comparison test. (mice: n ≥ 8) *NS*: non-significant, *P < 0.05, **P < 0.01

Masson’s trichrome staining was further performed to visualize collagen compositions in the wound regions from different groups at day 15. It was found that the wound regions from RCM-fiber-BMMSCs treated group have less total collagen deposition than those from Un-fiber-BMMSCs treated group (Fig. 3A), whereas there were more collagen depositions in the wound regions upon the treatments of Un-fibers, RCM-fibers or Un-fiber-BMMSCs. We also performed Picrosirius red staining to discriminate between mature and immature collagens depositing in the wound areas. Immature collagen (regarded as Collagen Type 3) was labeled in green/yellow while organized and mature collagen fibers (regarded as Collagen Type 1) were labeled in orange/red. RCM-fiber-BMMSCs treated wounds were largely composed of green/yellow collagen fibers as immature collagens and intermixed in a basket weave orientation indicating normal healing processes. On the contrary, in the wounds from Un-fibers, the RCM-fibers or the Un-fiber-BMMSCs treated groups, orange/red collagen fibers were dominant with parallel orientation implying the fibrosis of local wound regions (Fig. 3B). When calculating the percentages of immature and mature collagens in different groups, it was found that there were the lowest percentages of mature Collagen 1 and the highest percentages of immature Collagen 3 in the wound regions treated with the RCM-fiber-BMMSCs living material (Fig. 3C).

It is recognized that BMMSCs alone are still vulnerable to oxidative stress during wound healing. Our in vitro assays demonstrated that the RCM-fiber-BMMSCs possessed more resistance to H_2_O_2_-mediated oxidative stress with better proliferation capacity. To evaluate the efficacy of the RCM-fiber-BMMSCs in the resistance to oxidative stress in diabetic wound therapy, we stained the wound skins with 8-OHdG, a biomarker for DNA oxidative damage. It was found that the wounds treated with the RCM-fiber-BMMSCs had less 8-OHdG staining than those either with the Un-fiber-BMMSCs or other groups on day 7 (Fig. 3D, i). In addition, neovascularization in the skin wounds is also one of the key therapeutic objectives during wound healing. It was apparent that higher proportions of CD31^+^ cells, an indicator for angiogenesis in the wound areas, were observed upon the treatment of the RCM-fiber-BMMSCs than those receiving other treatments at day 7 (Fig. 3D, iii), demonstrating improved angiogenesis ability of the RCM-fiber-BMMSCs. By using CK10 staining (a differentiation marker for keratin), we further validated better re-epithelialization with more CK10 expressions in the wound areas from RCM-fiber-BMMSCs treated mice when compared to those from other groups (Fig. 3D, ii). Collectively, these results support that the RCM-fiber-BMMSCs accelerate diabetic wound closure accompanied by rapid re-epithelialization largely through collagen remodeling and accelerating angiogenesis in the wound areas.

### RCM-fiber-BMMSCs treatment leads to decreased inflammation in diabetic wound regions

**Fig. 4:**
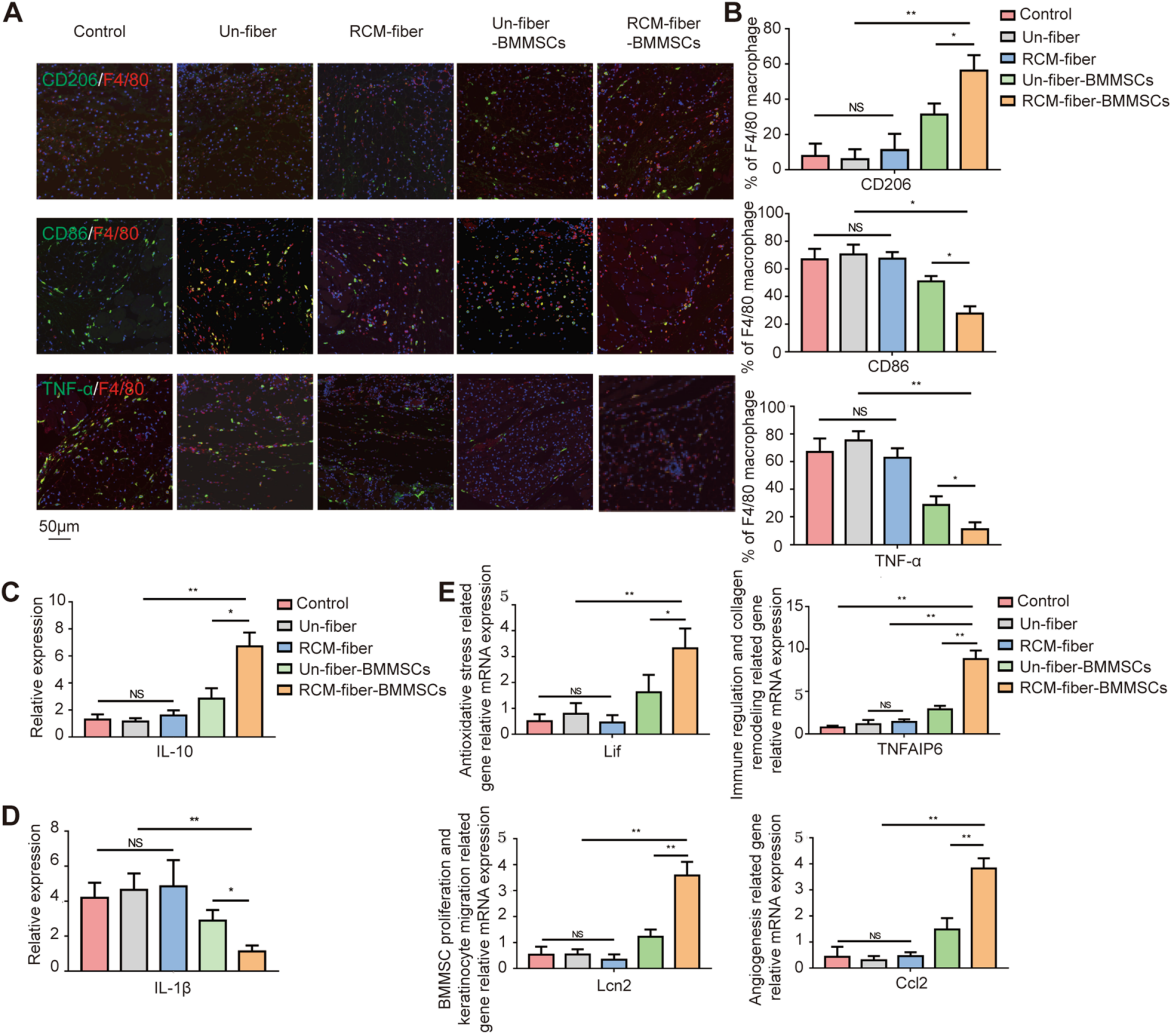
RCM-fiber-BMMSCs treatment leads to decreased inflammation in diabetic wound regions.** A** Immunofluorescence staining for F4/80 (red), CD206 (green), CD86 (green) and TNF-α (green) in the wound tissues. Scale bar = 50 μm. **B** Comparisons of the proportions of F4/80^+^CD86^+^, F4/80^+^TNF-α^+^ and F4/80^+^CD206^+^ cells in the wound areas among different groups. **C**, **D** Expression levels of *IL-1β* (**C**) and *IL-10* (**D**) in diabetic cutaneous wound areas on day 7 by real-time PCR. **E** Relative expression levels of *Ccl2*, *Lif*, *TNFAIP6* and *Lcn2* in the diabetic skin wounds treated with different nanofibers. Data were represented as mean ± SD. Differences were analyzed by using one-way ANOVA followed by Tukey’s multiple comparison test. (mice: n ≥ 8) *NS*: non-significant, *P < 0.05, **P < 0.01

Long-term excessive inflammation prolongs the healing processes of diabetic wounds. We further investigated whether the RCM-fiber-BMMSCs also reduced the inflammatory responses during diabetic wound repair. We found that the percentages of F4/80^+^CD86^+^ macrophages have been decreased in accordance with the lowest percentages of F4/80^+^TNF-α^+^ cells in the wound areas upon RCM-fiber-BMMSCs treatment. On the contrary, the percentages of F4/80^+^CD206^+^ macrophages were the highest on day 7 post-wounding when receiving RCM-fiber-BMMSCs treatment (Fig. 4A and B). We also determined the expression levels of *IL-1β* (a vital inflammatory cytokine) and *IL-10* (an immunosuppressive cytokine) in the wound regions by RT-PCR. It was found that *IL-1β* expression was prominently reduced in the wound tissues on day 7 after RCM-fiber-BMMSCs treatment (Fig. 4C) whereas the level of *IL-10* was exaggerated (Fig. 4D). These data demonstrate that RCM-fiber-BMMSCs treatment is inclined to suppress local inflammation with more extent through the suppression of inflammatory cytokine production and the induction of inhibitory cytokine IL-10.

Being a promising therapeutic tool, BMMSCs have been demonstrated to express extensive bioactive mediators for tissue repair in vivo, among which tumor necrosis factor-induced protein 6 (TNFAIP6) [25, 31], Lipocalin-2 (Lcn-2) [32–34], chemokine (C-C motif) ligand 2(Ccl2) [35–37] and leukaemia inhibitory factor (Lif) [38–40] exert multiple functions including cell survival, immunomodulation, keratinocyte migration, angiogenesis and resistance to oxidative stress respectively. Therefore, expression levels of four genes were detected by RT-PCR in the wound regions at the early time of the treatments. All four genes displayed remarkably increased expressing levels in the wounds when treated with the RCM-fiber-BMMSCs compared to the Un-fiber-BMMSCs, whereas the wounds from the mice treated with the fibers without loading BMMSCs expressed low levels of four genes (Fig. 4E). These results further indicate that the RCM-fiber-BMMSCs induce less inflammatory environments in the wound areas, which is largely associated with accelerating diabetic wound healing.

### The RCM-fibers promote the expressions of wound healing related genes in BMMSCs facilitating keratinocyte migration and less inflammation

**Fig. 5:**
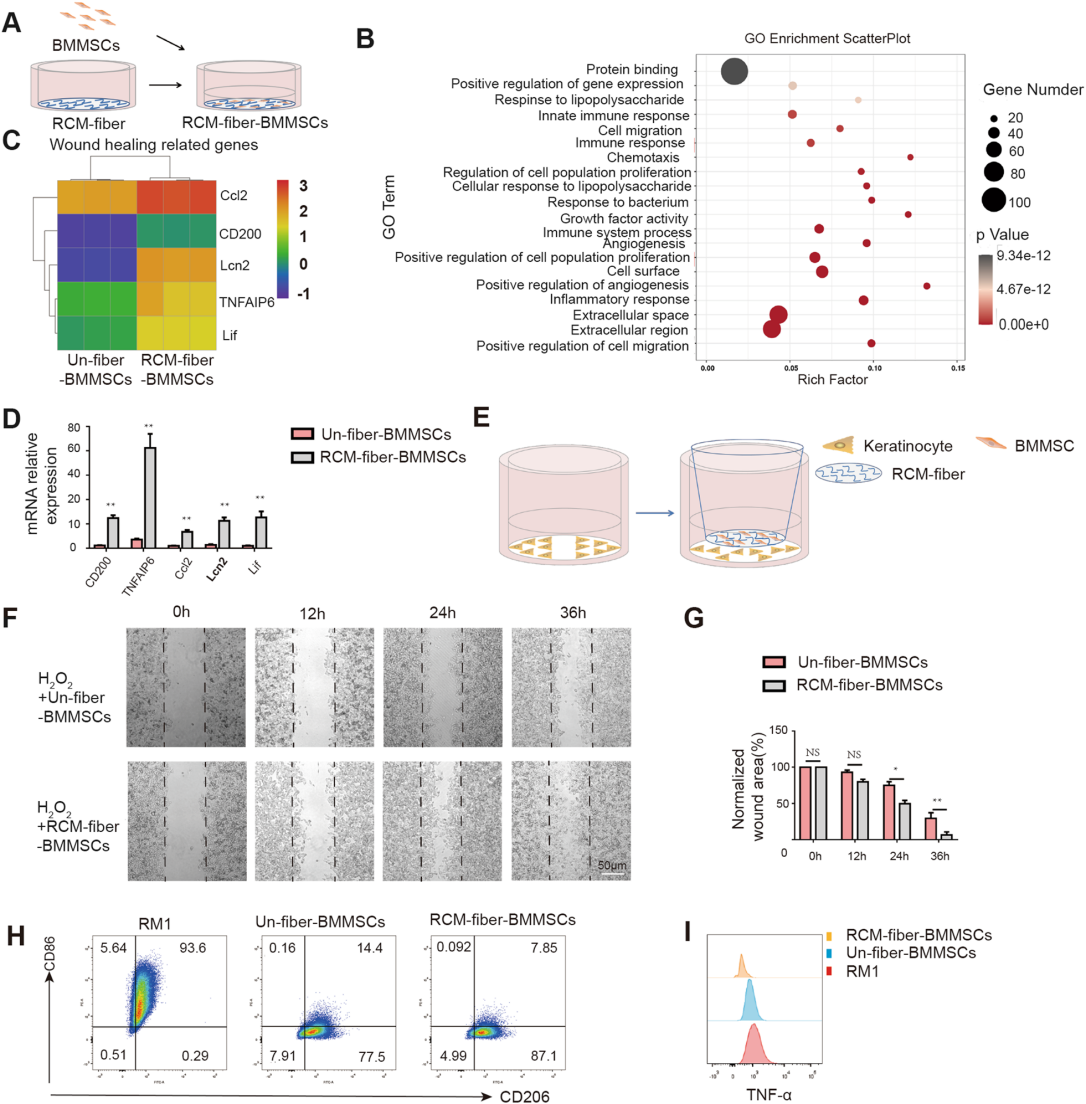
RCM-fibers promote the expressions of wound healing related genes in BMMSCs facilitating keratinocyte migration and less inflammation.** A** Schematic illustration showing the cocultures of BMMSCs with the RCM-fibers in vitro. **B** Gene ontology enrichment scatter plots in key signaling pathways. **C** Heat map analysis of wound healing related gene expressions in BMMSCs after coculturing with either the Un-fibers or the RCM-fibers for 48 h. **D** Validation of expression levels of *CD200*, *Ccl2*, *Lif*, *TNFAIP6* and *Lcn2* in BMMSCs after coculturing with either the Un-fibers or the RCM-fibers for 48 h by real-time PCR. **E** Schematic illustration on the migration of JB6 cell incubated with either the RCM-fiber-BMMSCs or the Un-fiber-BMMSCs upon H_2_O_2_ treatment. **F** Representative images of the wound areas covered by JB6 cell at 12, 18, 24 and 36 h. Scale bar = 50 μm. **G** Comparisons of normalized wound areas covered by JB6 cell at different time points. **H** CD86 and CD206 expressions on LPS/IFN-γ activated RAW264.7 cells after incubating with the RCM-fiber-BMMSCs or the Un-fiber-BMMSCs. **I** TNF-α expression in LPS/IFN-γ activated RAW264.7 cells after incubating with the RCM-fiber-BMMSCs or the Un-fiber-BMMSCs. Data were represented as mean ± SD. Differences were analyzed by one-way ANOVA with Tukey’s multiple comparison test. The experiments were repeated in triplicates. *P < 0.05, **P < 0.01

To define molecular signatures of BMMSCs regulated by the RCM-fibers, BMMSCs were cocultured with either the RCM-fibers or the Un-fibers in vitro and the transcriptomes were compared by using RNA-sequencing (seq) (Fig. 5A). Based on the RNA-seq results, there were 449 genes upregulated and 275 genes down-regulated in BMMSCs cocultured with the RCM-fibers when compared to those with the Un-fibers. GO enrichment scatter plot analysis revealed that the genes related to immunoregulation, cell proliferation, angiogenesis and cell migration were increased dramatically in BMMSCs cocultured with the RCM-fibers (Fig. 5B). Consistent with the results from in vivo wound samples, four wound healing related genes we detected in the wound areas including *Ccl2*, *Lif*, *TNFAIP6* and *Lcn2* were more highly expressed in RCM-fiber-cocultured BMMSCs than those with Un-fibers (Fig. 5C), which was also validated by RT-PCR (Fig. 5D).

Furthermore, we performed the in vitro assays to verify the influence of the RCM-fiber-BMMSCs on keratinocyte migration. Mouse keratinocyte cell line JB6 was cocultured with the RCM-fiber-BMMSCs or the Un-fiber-BMMSCs in the transwells with the addition of H_2_O_2_ to mimic oxidative stress (Fig. 5E). To avoid the interference of cell proliferation, JB6 cells were starved in serum-free culture medium for 24 h before the injury. It was found that the migration of JB6 cells with the RCM-fiber-BMMSCs was faster than those with the Un-fiber-BMMSCs. Within 36 h the injury has nearly covered by JB6 cells incubated with the RCM-fiber-BMMSCs whereas the injury was still open in the Un-fiber-BMMSCs group (Fig. 5F and G), which indicate that the RCM-fibers enhance BMMSCs’ ability to promote keratinocyte migration under oxidative stress.

We also compared the effects of the RCM-fiber-BMMSCs and the Un-fiber-BMMSCs on macrophage differentiation in vitro (Fig. 5H). LPS and IFN-γ treated RAW264.7 cells exhibited dramatic upregulation of CD86 and the reduction of CD206 (named as RM1) (Fig. 5I, left). Incubation of RM1 cells with either the RCM-fiber-BMMSCs or the Un-fiber-BMMSCs dramatically reduced the expressions of CD86 and maintained CD206 expressions (Fig. 5I, middle and right). From in vitro investigations, we validate gene signatures of BMMSCs regulated by the RCM-fibers most of which are associated with wound healing. The RCM-fibers therefore provide supportive roles for BMMSCs in promoting keratinocyte migration as well as reducing the inflammation.

### CD200R-CD200 ligation is engaged in accelerating diabetic wound healing by the RCM-fiber-BMMSCs

**Fig. 6:**
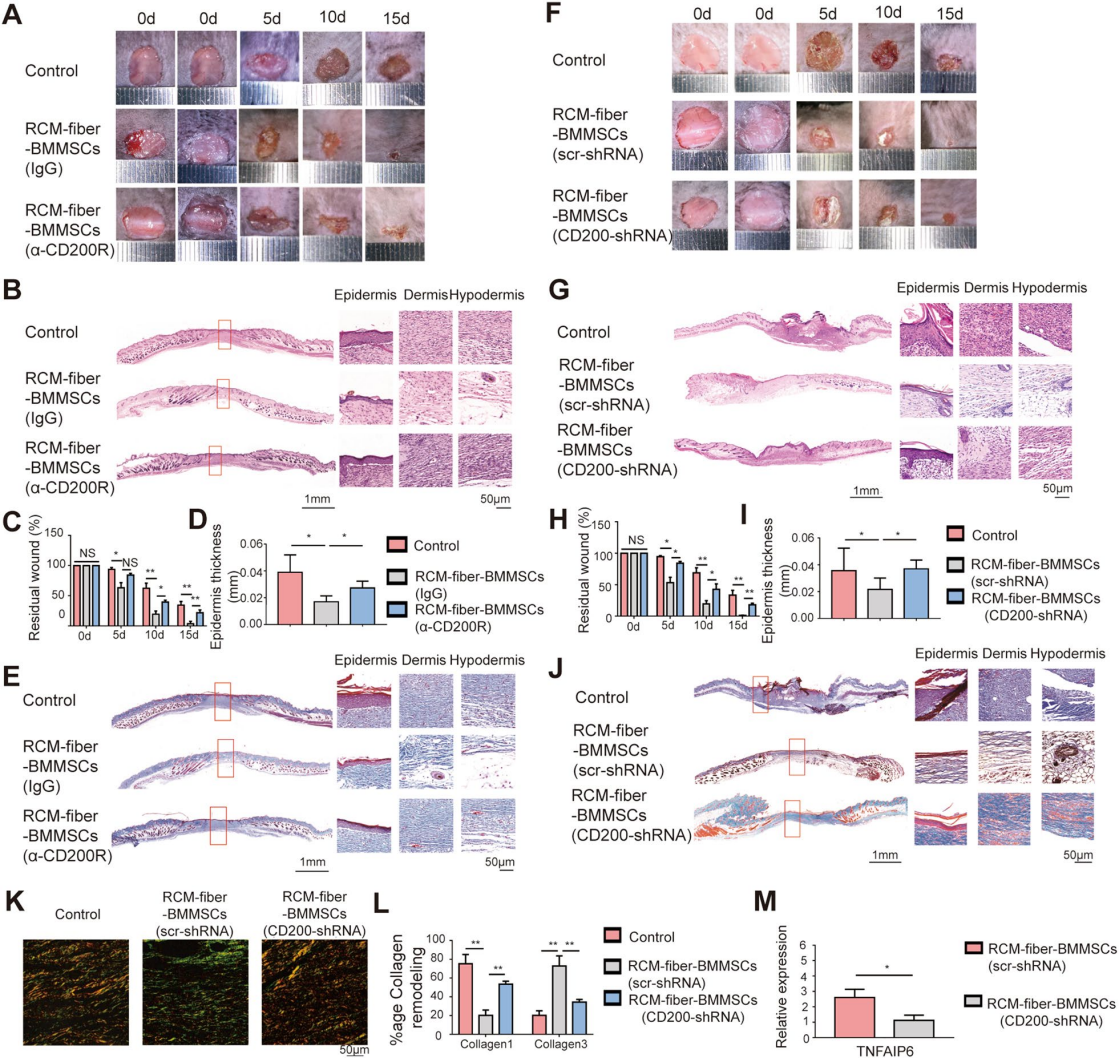
CD200R-CD200 ligation is engaged in accelerating diabetic wound healing by the RCM-fiber-BMMSCs. **A** Representative images of the wound areas at day 0, 5, 10 and 15 post-wounding upon anti-CD200R or isotype IgG treatment. **B** Representative images of H&E staining for the wound regions at day 15 (left) with high-resolution images of epidermis, dermis and hypodermis (right) upon RCM-fiber-BMMSCs treatment with the presence of anti-CD200R or isotype IgG. Scale bar = 1 mm (left) or 50 μm (right). **C** Comparisons of residual wound areas at each time point upon RCM-fiber-BMMSCs treatment with the presence of anti-CD200R or isotype IgG. **D** Comparisons of average epidermal thickness of the wounds upon RCM-fiber-BMMSCs treatment with the presence of anti-CD200R or isotype IgG. **E** Images of Masson’s trichrome staining for the wound tissues at day 15 (blue for collagen). Scale bar = 1 mm (left) or 50 μm (right). **F** Representative images of the wound areas at day 0, 5, 10 and 15 upon RCM-fiber-BMMSCs treatment with or without *CD200* expressions in BMMSCs. **G**, **H** Representative images of H&E staining (**G**) for the wound areas at day 15 (left) with high-resolution images of epidermis, dermis and hypodermis (right) upon RCM-fiber-BMMSCs treatment with or without *CD200* expressions in BMMSCs and comparisons of residual wound areas at each time point (**H**). Scale bar = 1 mm (left) or 50 μm (right). **I** Comparisons of average epidermal thickness of the wounds upon RCM-fiber-BMMSCs treatment with or without *CD200* expressions in BMMSCs at day 15. **J** Images of Masson’s trichrome staining for the wound tissues at day 15 (blue for collagen) upon RCM-fiber-BMMSCs treatment with or without *CD200* expressions in BMMSCs. Scale bar = 1 mm (left) or 50 μm (right). **K** Picrosirius red staining for collagen deposition in the wound areas. Scale bar = 50 μm. **L** Comparisons of the proportions of Collagen 1 and Collagen 3 in diabetic wound areas upon *CD200* interference in BMMSCs. **M** The levels of *TNFAIP6* in the wound tissues upon RCM-fiber-BMMSCs treatment with or without *CD200* expressions in BMMSCs. Data were represented as mean ± SD. Differences were analyzed through one-way ANOVA with Tukey’s multiple comparison test. (mice: n ≥ 8) NS: non-significant, *P < 0.05, **P < 0.01

From the RNA-seq analysis, it was found that *CD200* was dramatically upregulated on BMMSCs when incubated with the RCM-fibers (Fig. 5C and D). To define whether CD200-CD200R ligation is involved in the therapeutic effects of the RCM-fiber-BMMSCs on diabetic wound repair, we incubated the RCM-fiber-BMMSCs with anti-CD200R blocking antibody and applied to wound healing in diabetic mice. It was found that the incubation of anti-CD200R antibody with the RCM-fiber-BMMSCs slowed down the residual wound healing rates when compared to isotype control group (Fig. 6A). At day 15, the wound areas were 22.18 ± 4.01% with anti-CD200R treatment, which was significantly higher than those with isotype control treatment (3.98 ± 3.40%) (Fig. 6C). H&E staining of skin wounds at day 15 also demonstrated that there was more mature epidermis in RCM-fiber-BMMSCs group upon anti-CD200R treatment with the increase in epidermis thickness when compared to isotype control treatment (Fig. 6B, D). In addition, the results from masson’s trichrome staining also revealed more collagen deposition in anti-CD200R plus RCM-fiber-BMMSCs treated tissues at day 15 when compared to isotype IgG plus RCM-fiber-BMMSCs treated wounds (Fig. 6E).

In parallel, we have knocked down *CD200* expressions in BMMSCs with shRNA and prepared the RCM-fiber**-**BMMSCs(*CD200KO*) to validate the impacts of CD200-CD200R ligation on wound healing. Consistent with the results from anti-CD200R blocking assays, RCM-fiber**-**BMMSCs(*CD200KO*) treatment also slowed down wound closure when compared to RCM-fiber**-**BMMSCs treatment (Fig. 6F) with more residual wounds at day 5, day 10 and day 15 (Fig. 6H). There also displayed more epidermis thickness and collagen deposition at day 15 in RCM-fiber-BMMSCs treated wound regions with *CD200* deficiency when compared to control BMMSCs (Fig. 6G–J). Results from picrosirius red staining showed that the wounds upon RCM-fiber-BMMSCs(*CD200KO*) treatment had more Collagen 1 deposition and less Collagen 3 deposition when compared to the RCM-fiber-BMMSCs group (Fig. 6K and L), which was similar to anti-CD200R treatment (Additional file 1: Fig. S2). The expression levels of *TNFAIP6* were lower in the wound regions upon RCM-fiber-BMMSCs (*CD200KO*) treatment than the counterpart group (Fig. 6M). Based on the results from anti-CD200R blocking assays and knockdown of *CD200* in BMMSCs, we conclude that CD200R-CD200 ligation is largely engaged in accelerating diabetic wound healing mediated by the RCM-fiber-BMMSCs.

### LPS/IFN-γ activated THP-1 cell membrane-modified nanofiber scaffolds improve the effects of human BMMSCs in diabetic skin wound healing

**Fig. 7:**
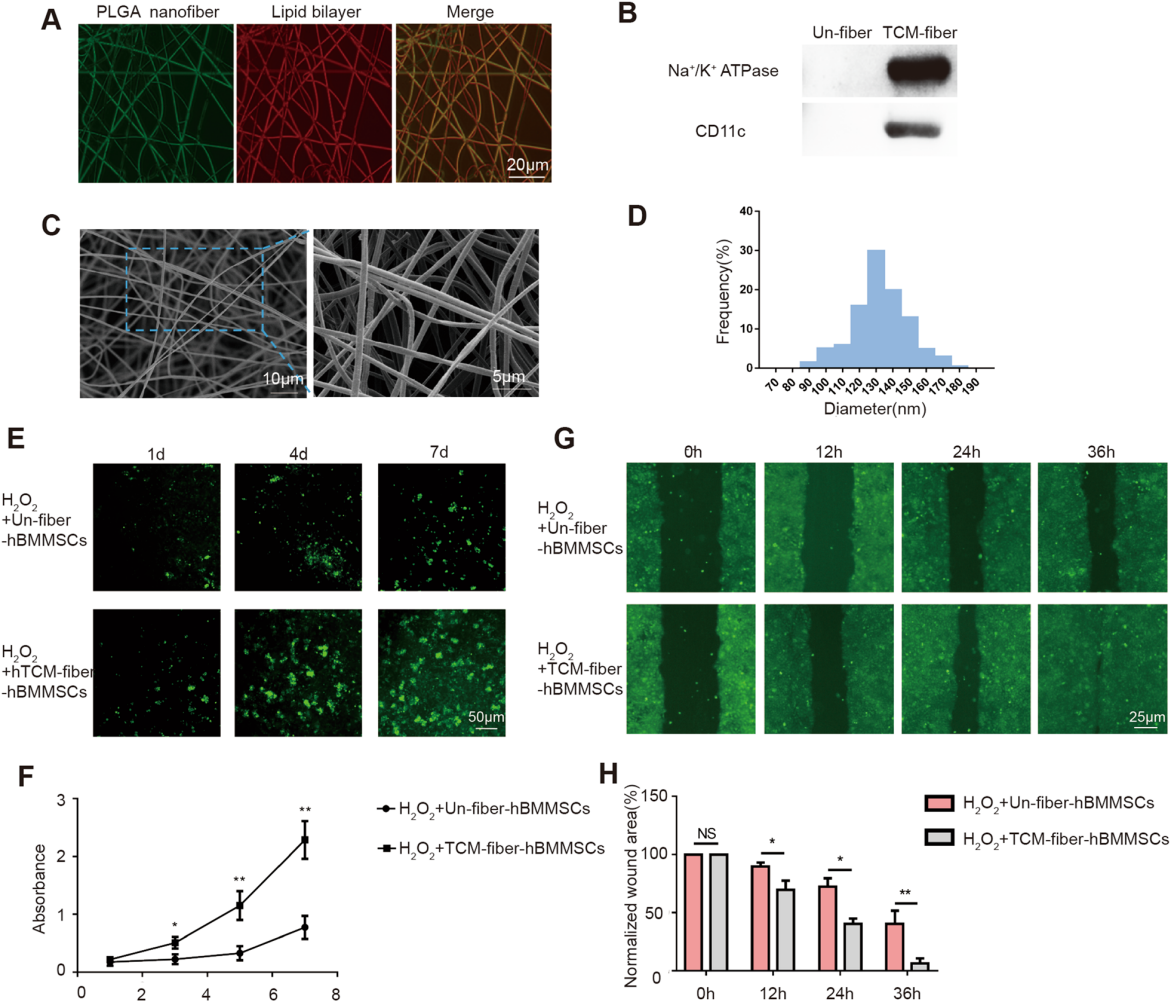
Physiochemical and biological characterization of the TCM-fibers.** A** Representative fluorescent images of the TCM-fibers after coumarin (for PLGA nanofiber) and Dil (for lipid bilayer) staining. Scale bar = 20 μm. (**B** Detection of Na^+^/K^+^ ATPase and CD11c proteins on the TCM-fibers and Un-fibers (without cell membrane) by Western blotting. **C** Representative images of TCM-fibers morphology by a scanning electron microscopy. Scale bar = 10 μm (left) or 5 μm (right). **D** Size distribution of the TCM-fibers. **E** Fluorescent images of human BMMSCs (hBMMSCs) cultured on the Un-fibers or RCM-fibers by CFDA-SE staining. Scale bar = 5 μm. **F** CCK-8 assays for cell proliferation of hBMMSCs at day 1, day 3, day 5 and day 7, respectively. **G** Representative images of the wound areas covered by HaCaT cells at 12, 24 and 36 h in cell migration assay exposed to H_2_O_2_ in vitro. Scale bar = 25 μm. **H** Comparisons of the percentages of the residual wound areas covered by HaCaT cells at different time points normalized to the original would areas. Data were represented as mean ± SD. Differences were analyzed by one-way ANOVA with Tukey’s multiple comparison tests.(n ≥ 3) *P < 0.05, **P < 0.01

Our aforementioned results indicated that mouse-derived RCM-fiber-BMMSCs were apparently able to ameliorate wound healing processes. We further modified the nanofibers with cell membrane from human derived THP-1 cells pre-treated with LPS and IFN-γ to prepare the TCM-fibers. Similar to the RCM-fibers, when cell membrane from activated THP-1 was scattered on the nanofibers confirmed by coumarin (green) and Dil (red) staining (Fig. 7A), Na^+^/K^+^-ATPase and CD11c proteins were detected only on the TCM-fibers rather than on the Un-fibers (Fig. 7B). Based on SEM detection, we found that the TCM-fibers also had a smooth outer surface and long fibrous morphology (Fig. 7C) and the fibers loaded with cell membrane had the diameters mainly in the range of 100–150 nm (Fig. 7D). When we incubated human BMMSCs (hBMMSCs) with either the TCM-fibers or the Un-fibers exposed to H_2_O_2_ at different times in vitro, more green fluorescence derived from CFDA-SE staining was detected on day 4 and day 7 in hBMMSCs cocultured with the RCM-fibers when compared to those with the Un-fibers (Fig. 7E). Based on the CCK-8 assay, the TCM-fibers also promoted the proliferation of hBMMSCs when compared to the Un-fibers (*P* < 0.05) (Fig. 7F), further demonstrating similar roles of the TCM-fibers in promoting the proliferation of hBMMSCs.

**Fig. 8:**
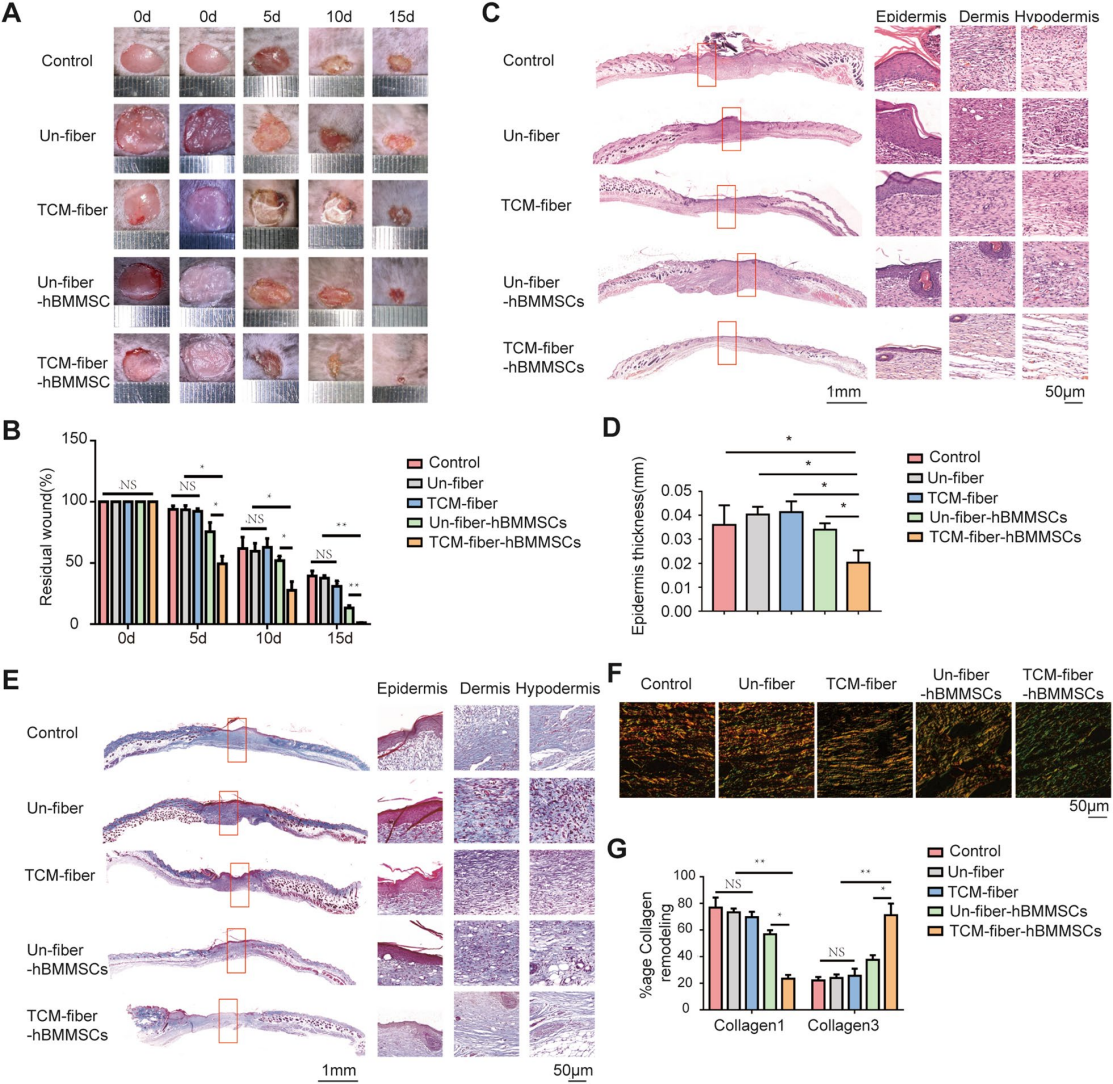
TCM-fiber-hBMMSCs improve wound healing in diabetic mice. **A** Representative images of the wound areas at day 0, 5, 10 and 15 post-wounding, respectively. **B** Quantification of residual wound areas at day 0, 5, 10 and 15, respectively. **C** Representative images of H&E staining for the wound areas at day 15 (left) with high-resolution images of epidermis, dermis and hypodermis (right). Scale bar = 1 mm (left) or 50 μm (right). **D** Quantification of average epidermal thickness of the wound areas. **E** Masson’s trichrome staining for tissue sections (left) with high-resolution images of collagen deposition in the epidermis, dermis and hypodermis (right) in diabetic mouse wounds. Scale bar = 1 mm (left) or 50 μm (right). **F** Picrosirius red staining for collagen deposition in the wound areas from diabetic mice. Scale bar = 50 μm. **G** Comparisons of Collagen 1 and Collagen 3 proportions in the wound areas among different groups. Data were represented as mean ± SD. Differences were analyzed through two-way and one-way ANOVA with Tukey’s multiple comparison tests. (mice: n ≥ 8) *NS*: non-significant, *P < 0.05, **P < 0.01

Electrospun nanfibers loading hBMMSCs were prepared as another living material named as the TCM-fiber-hBMMSCs. We found that the TCM-fiber-BMMSCs accelerated the migration of HaCaT cells when compared to those with the Un-fiber-hBMMSCs in the in vitro wound healing test even exposed to H_2_O_2_ (Fig. 7G, H). The effects of the TCM-fiber-hBMMSCs on diabetic wound healing were further evaluated. It was found that the TCM-fiber-hBMMSCs also accelerated wound closure with the smallest residual wound areas especially at day 15 when compared to other groups (Fig. 8A, B). Results from H&E staining indicated that the differentiation of epidermal cells and the formation of hair follicles were enhanced upon the treatment of the TCM-fiber-hBMMSCs (Fig. 8C and D). In the wound regions there showed faster collagen deposition (Fig. 8E) with increased Collagen 3 and less Collagen 1 in the wound regions from TCM-fiber-hBMMSCs treated group (Fig. 8F and G) when compared to other groups. Therefore, similar to the RCM-fiber-BMMSCs, the TCM-fiber-hBMMSCs living material also promotes wound healing with the prevalent significance.

## Discussion

In the present study, we have modified the nanofibers with LPS/IFN-γ activated macrophage cell membrane which is demonstrated to promote the proliferation of BMMSCs and enhance the resistance to oxidative stress. BMMSCs loading on cell membrane-modified nanofibers accelerate wound closure in diabetic mice. LPS/IFN-γ-activated macrophage cell membrane modified electrospun nanofibers loaded with BMMSCs become an alternative potential immuno-activated living material to potentiate the clinic applications of BMMSCs in the future.

Biomaterials have been widely used in tissue regeneration. An ideal biomaterial possesses essential properties such as hierarchical architectures and ECM composition similar to native tissues [41]. Nanofibers have been widely used in regenerative engineering due to their feasibility in modification, more interactions with tissue environment as well as delivery of regenerative signals to the tissues [42]. Among multiple modifications, cell membrane modified nanofibers exhibit more advantages such as strong hydrophilicity, high velocity of degradation and fruitfulness of *bona fide* surface molecules anchoring on living cells [30]. In our study, we used LPS/IFN-γ activated macrophage cell lines-derived cytomembrane to modify the nanofibers. Both mouse RAW264.7 and human THP-1 cells belong to monocyte lineage that can be easily activated to different macrophage subtypes based on conditional cytokines such as LPS/IFN-γ for M1-like macrophages and IL-4 for M2-like macrophages [43]. In fact macrophage-MSC crosstalk generates paracrine signals to affect cell differentiation which is related to the outcomes of tissue regeneration. For instance, exosomes from the MSCs can affect the differentiation to either inflammatory (M1) or regenerative (M2) macrophages [44]. Activated M1 or M2-like macrophages can also secrete multiplex bioactive molecules to guide MSC differentiation and affect their survival and proliferation [45]. We have used LPS/IFN-γ activated macrophage cell membrane to modify the nanfibers because previous studies have already demonstrated the efficacy of M1-like macrophages to support the survival and proliferation of BMMSCs. In our study, *TNFAIP6* could be induced in BMMSCs when they were loaded on LPS/IFN-γ activated macrophage cell membrane-containing nanofibers whereas little was detectable in BMMSCs with nanofibers only or with IL-4 activated macrophage cell membrane-containing nanofibers (Additional file 1: Fig. S3). This further supports the roles of M1-like macrophages in modulating regenerative properties of BMMSCs.

What is more, based on our results the nanofibers modified with LPS/IFN-γ activated macrophage cell membrane exaggerate the resistance of BMMSCs to oxidative stress. When BMMSCs are transplanted to the wound regions where hypoxic environments are usually generated with increased reactive oxygen species (ROS) [46]. There usually occurs the inhibition of the proliferation and immunoregulatory roles of BMMSCs [47] whereas the increase in the senescence and adipogenic differentiation of BMMSCs [48]. In our study, the RCM-fibers and the TCM-fibers not only favor BMMSC proliferation in oxidative stress, but also promote the migration of keratinocytes in vitro. This was consistent with rapid re-epithelialization and wound closure in diabetic mice. LPS/IFN-γ activated macrophage cell membrane-modified nanofibers thus largely augment the resistance of BMMSC to harmful microenvironments.

When the RCM-fibers were used to load BMMSCs (RCM-fiber-BMMSCs) for in vivo diabetic wound healing in mice, more advantages were shown up including epidermal formation, collagen remodeling and angiogenesis. These events are all key steps for skin regeneration which are also interacted. Firstly, keratinocyte activation and differentiation are strongly associated with epidermal formation [49]. In our study we have observed incomplete differentiation of keratinocytes in the Un-fibers, RCM-fibers and Un-fiber-BMMSCs treated wounds. Keratinocyte migration is also accelerated in RCM-fiber-BMMSCs treated wounds leading to complete differentiation within the speculated time period. Secondly, the RCM-fiber-BMMSCs also promote rapid accumulation of epidermal cells in the wound areas developing mature epithelial structures such as hair follicles, making skin regeneration more rapid. Thirdly, during initial wound healing period there is increased fibroblast proliferation followed by the deposition of immature Collagen 3, which is mainly applied for wound closure to avoid further damage [50, 51]. Collagen remodeling and collagen composition in the wound areas are also key features to evaluate the regeneration of the wound regions. In this study, we have observed less total collagen and chiefly immature collagen deposition in the wound areas when treated with the RCM-fiber-BMMSCs. Fourthly, we have also observed more angiogenesis in the wound areas upon RCM-fiber-BMMSCs treatment, which indicates that the RCM-fiber-BMMSCs might overcome defective angiogenesis in diabetic wounds mostly due to enhanced mitochondrial ROS production [46]. All these data strongly support that the RCM-fibers remodel BMMSCs with more features to promote diabetic wound regeneration. The RCM-fiber-BMMSCs thus display the advantages in maintaining cell functionality and remodeling wound environment, making this living material more promising in wound repair.

To further define the molecular signatures of BMMSCs induced by the RCM-fibers, we have performed the RNA-seq to compare gene expression profiles of BMMSCs upon RCM-fibers or Un-fibers treatments. It was found that the expressions of *Ccl2*, *Lif*, *TNFAIP6* and *Lcn2* were dramatically increased both in vitro and in vivo. These four genes are already demonstrated to be engaged in skin regeneration. TNFAIP6 regulates immunological function and accelerates collagen remodeling [25, 31]. Lcn2 promotes BMMSC proliferation in oxidative stress and keratinocyte migration [32–34]. Lif protects against diabetic wound from oxidative stress [38–40]. Ccl2 accelerates the vascularization in the wound regions [35–37]. The increase in the expressions of four genes in RCM-fiber-loaded BMMSCs is extremely consistent with high efficacy of wound closure and skin regeneration in diabetic mice. The in situ immunostimulation on BMMSCs by cell membrane both in vitro and in vivo largely favors the biofunctionality of this living material.

Moreover, from RNA-seq analysis we have identified the upregulation of *CD200* in BMMSCs that was strongly associated with RCM-fiber-BMMSCs mediated wound healing. Both anti-CD200R treatment and silencing *CD200* in BMMSCs restrain the wound healing effects when compared to RCM-fiber-BMMSCs treatments. Recent research revealed that direct contact with M1-type macrophages not only increased the production of TNFAIP6 but also upregulated CD200 expression on BMMSCs [25]. CD200 is widely expressed on DC, thymocytes, B cells, MSCs, the central nervous system nerves, *etc* [52]. It can interact with its ligand CD200R to trigger the recruitment and phosphorylation of Dok-1/2, and further binds to RasGAP [53]. In macrophages and mast cells, this cascade has been shown to inhibit the phosphorylation of ERK, P38, and JNK signaling and interrupt the activation of myeloid cells [54]. Although we did not determine cell types expressing CD200R in wound regions, the regulatory roles of CD200-CD200R signaling defined in other cell types are consistent with the observations that RCM-fiber-BMMSCs treatment leads to low inflammation in the wound areas with the upregulation of CD200 in the wound regions. The exact mechanisms will be investigated further.

Last but not the least, we have also demonstrated the efficacy of human BMMSCs in promoting diabetic wound healing in mice after loading on electrospun nanofibers modified with LPS/IFN-γ activated THP-1 macrophage cytomembrane. Human BMMSCs can be either injected topically or mixed with various biomaterials including nanofiber scaffolds, hydrogels, extracellular matrix derivatives and dermal substitutes [55, 56]. They are demonstrated to promote skin wound healing by multiple mechanisms including releasing bioactive molecules, reducing the inflammation, stimulating resident cells and remodeling extracellular matrix [57]. When loaded on the TCM-fibers, hBMMSCs have accelerated the repair processes of diabetic wounds, which is similar to mice-derived RCM-fiber-BMMSCs. We have also observed the improvement of the TCM-fibers on hBMMSCs proliferation (Fig. 7). The TCM-fiber-hBMMSCs have promoted human keratinocyte migration under oxidative stress (Fig. 7G, H). Cell membranes derived from both human and mice LPS/IFN-γ activated macrophages modulate BMMSCs with similar patterns. Similar therapeutic effects of the RCM-fiber-BMMSCs and the TCM-fiber-hBMMSCs living materials on diabetic wound healing to some extent validate the universality of M1-like macrophage cell membrane modified nanofibers as an alternative scaffolds for BMMSCs in clinical application of wound repair in the future.

## Conclusions

Collectively, we have prepared the nanofibers electrospun with LPS/IFN-γ activated macrophage cell membrane to load human or mice BMMSCs as a novel living material for diabetic skin regeneration. Our results reveal that cell membrane coated electrospun nanofibers not only augment the proliferation of BMMSCs under oxidative stress in vitro, but also accelerate wound healing processes in diabetic mice through favoring immunoregulation, keratinocyte migration, collagen remodeling, antioxidant stress and neovascularization in the wound regions. This is in part due to the upregulation of CD200 on BMMSCs induced by cell membrane-loaded nanofibers, leading to CD200R-CD200 ligation and subsequently gene expressions. We have thus provided an alternative engineering way of nanofiber scaffolds and developed a novel living material with in situ immunostimulation capacity for the improvement in diabetic chronic wound management.

## Materials and methods

### Cell culture

Human bone marrow-derived mesenchymal stem cells (hBMMSCs) and mouse BMMSCs were purchased from Cyagen Biosciences Co., Ltd (Guangzhou, China). Mouse macrophage cell line RAW264.7, mouse keratinocyte cell line JB6, human macrophage cell line THP-1 and human epithelial cell line HaCaT cell line were purchased from Beina Biotechnology Co., Ltd (Guangzhou, China). All cells were maintained following the company’s instructions. For in vitro activation, RAW264.7 cells were treated with 1 µg/mL LPS (Sigma-Aldrich) and 20 ng/mL IFN-γ (Sigma-Aldrich) for 12 h. THP-1 cells were treated with 1 µg/mL LPS (Sigma-Aldrich) and 20 ng/mL hIFN-γ (Sigma-Aldrich) for 24 h after pre-treatment with 200 ng/mL Phorbol-12-myristate-13-acetate (PMA) (Sigma-Aldrich) for 6 h.

### Preparation and characterization of cell membrane-modified PLGA nanofibers

PLGA balls containing PLA and polyglycolide (PGA) at the ratio of 85%:15% were dissolved in dimethylformamide and tetrahydrofuran. The PLGA copolymer solution was loaded into a 5 mL syringe. An electric field was generated between the anode and the rectangular stainless steel plate with an 18 kV high voltage power supply. The PLGA copolymer solution was then ejected from the syringe under the accurate infusion control pump and sprayed onto the stainless steel plate with tin foil paper through the electrostatic field [58].

RAW264.7 and THP-1 cells were activated by LPS and IFN-γ with the properties of the macrophages [59, 60]. LPS/IFN-γ-treated and untreated RAW264.7 or THP-1 cells were ultrasonicated and cell membrane was collected. Cell membrane was harvested as previously reported. Briefly, cells were grown in T-175 culture flasks to full confluency and detached by scrapping culture flask surfaces. Cells were suspended in a hypotonic lysing buffer containing 20 mM Tris-HCl (pH = 7.5), 10 mM KCl, 2 mM MgCl_2_ and EDTA-free protease inhibitor (Solarbio), and disrupted using a dounce homogenizer with a tight-fitting pestle. Cell suspensions were centrifuged at 20,000*g* for 20 min. The supernatants were collected and centrifuged again at 100,000*g* for 45 min. The pellets were collected containing cell membrane for subsequent experiments. Cell membrane suspension was added to the nanofibers and soaked for 30 min. The solvent was discarded and the nanofiber scaffolds were rinsed with PBS. The morphologies of the RCM-fibers (with LPS/IFN-γ-treated RAW264.7 cell membrane) and the TCM-fibers (with LPS/IFN-γ-treated THP-1 cell membrane) were detected by a scanning electron microscopy (SEM) (Carl Zeiss). The diameter and size distribution of the nanofibers were calculated by measuring at least 100 fibers which were selected randomly from five independent samples.

To characterize the PLGA nanofibers, 10 µL coumarin fluorescent dyes (1 µM) (Sigma-Aldrich) was added to PLGA copolymer solution according to the manufacturer’s instructions. 1 mL 1,1′-dioctadecyl-3,3,3′,3′tetramethyl-indocarbocyanine perchlorate (DiL) (5 µM) (Sigma-Aldrich) was added on 1 cm^2^ RCM-fibers, TCM-fibers and Un-fibers according to the product description. The water contact angles of modified nanofibrous membranes were measured using water contact angle analyzer (FTA200). The samples were put on the testing plate and a drop of distilled water was carefully placed on the surface of the samples. The measurements were carried out five times for each sample.

### Western blotting

The RCM-fibers and the TCM-fibers were decomposed by the ultrasound for 5 min with the addition of protease inhibitors. The proteins were collected and subjected to sodium dodecyl sulfate-polyacrylamide gel electrophoresis (SDS-PAGE). Proteins were then transferred to PVDF membrane (ThermoFisher Scientific). After blocking with phosphate buffer saline (PBS) containing 5% bovine serum albumin (BSA), the membranes were incubated with anti-mouse or anti-human CD11c (1:500) (Abcam) and anti-Na^+^/K^+^ ATPase (1:300) (Abcam) primary antibodies overnight at 4 °C. The membranes were further incubated with horseradish peroxidase (HRP)-conjugated secondary antibodies (1:2000) (Abcam). After washing four times with Tris buffer saline contain 0.5% Tween20 (TBS-T), immunopositive bands were visualized by chemiluminescence detection system (Merck Millipore).

### Flow cytometry

The cells were washed and incubated with fluorescence conjugated antibodies including PE-anti-mouse CD86 (Abcam) and FITC-anti-mouse CD206 (Abcam) for surface staining, and FITC-anti-mouse TNF-α antibody (Abcam) for intracellular cytokine staining according to the manufacturer’s instructions (BD Biosciences). Cells were washed, resuspended in PBS and acquired on BD FACSverse flow cytometer (BD Biosciences). The data were analyzed by Flowjo software (version 7.6.1) (Tree Star Inc.).

### Quantitative real-time PCR

Cells were mixed with TRIzol Reagent (Invitrogen). Total RNA was extracted and reversely transcribed into cDNA by using QuantiTect Reverse Transcription Kit (Qiagen) in accordance with the manufacturer’s instructions. Polymerase chain reaction (PCR) was performed by using SYBR Green qPCR Master Mix (MedChemExpress). Gene expression levels in individual samples were calculated based on the 2^−△△CT^ according to the threshold cycle (CT) values of target genes and house-keeping gene. The primers for target genes and *GAPDH* as the house-keeping gene were listed in Additional file 1: Table S1.

### Cell migration assay

Cell migration was determined by using a wound injury test in six-well transwell plates (Corning Inc.). After 24 h of cell attachment, JB6 or HaCaT cells were serum-starved overnight. To mimic oxidative stress, JB6 and HaCaT cells were exposed to 200 µM H_2_O_2_ for 2 h and continued to culture until more than 90% confluency. Cells were rinsed away by PBS to leave 0.5 cm-width vacancy and further incubated with the RCM-fiber-BMMSCs or the TCM-fiber-BMMSCs. Wound closure was imaged at 12 h, 24 and 36 h, respectively. The wound areas were quantified using ImageJ software to calculate the percentages of wound closure in vitro.

### BMMSCs proliferation assay

2 × 10^4^ third-generation mouse BMMSCs or hBMMSCs were seeded and exposed to 600 µM H_2_O_2_ for 2–6 h. BMMSCs were incubated with the RCM-fibers or the Un-fibers for 1, 4, and 7 days and stained with CFDA-SE (ThermoFisher Scientific) according to the manufacturer’s instructions. CCK-8 assay was also applied to determine the proliferation of BMMSCs on the RCM-fibers and the Un-fibers. After 1, 3, 5 or 7 days, the supernatant was discarded and 100 µL of CCK-8 solution was added to each well for another 4 h’s incubation. The OD values of each well were measured by an ELISA assay reader (Infinite M200) at 450 nm wave length.

### Knockdown of *CD200* in BMMSCs

BMMSCs were transfected with *CD200*-specific or nonspecific control short hairpin RNA (shRNA) (Ribobio Biotechnology) by using transfection reagent (Ribobio Biotechnology) according to the manufacturer’s protocol. The knockdown efficiency of *CD200* was detected by RT-PCR after 48 h.

### Surgical procedure

6-week-old C57BL/6J male mice were purchased from Tengxin Technology Co. Ltd and maintained under a specific pathogen-free (SPF) mouse facility. The mice were continuously fed on high-fat and high sugar diet for 4 weeks. On day 19, C57BL/6J mice were intraperitoneally injected with 100 µL streptozotocin (STZ) (50 mg/kg, Sigma-Aldrich) for 3 days. On day 28, blood glucose levels were surveyed using a glucometer (Sinocare, China). Diabetic mice were generated when blood glucose levels consistently exceeded 14 mM (Additional file 1: Fig. S1A and S1B).

Biopsy punches (1.0 cm diameter) were generated by removing epidermis and dermis. The Un-fibers, RCM-fibers, Un-fiber-BMMSCs and RCM-fiber-BMMSCs with similar diameter were applied on the wound beds while mice in the control group received no fiber treatment. Wound closure was determined through picture collection at day 0, 5 and 15 respectively, and analyzed by ImageJ software. In some experiments, anti-mouse CD200R (20 ng/mL) (Abcam) or IgG antibodies were mixed with the RCM-fiber-BMMSCs before subsequent experiments. In addition, BMMSCs with *CD200* deficiency were used in some experiments.

### Histological analysis and immunofluorescence assay

Mice were sacrificed after 15 days. The wound tissues were fixed in 10% formalin and embedded in the paraffin routinely. The sections of diabetic wounds were stained with hematoxylin and eosin (H & E), and Masson’s trichrome. Images were scanned by ServiceBio Company. Picrosirius red staining pictures were photographed by polarized light microscope in Servicebio Company. The collagen content was quantified using ImageJ v1.8.0 software.

For immunostaining, wound sections were dewaxed and rinsed with PBS. For antigen retrieval, tissue sections were treated with sodium citrate buffer at 95 °C for 10 min, followed by permeabilization with 0.1% Triton X-100 for 10 min. After blocking in PBST (PBS + 0.1% Tween 20) containing 2% BSA, tissue sections were incubated with 8-OHdG (Abcam; 1:200), anti-mouse CD31 (Abcam; 1:300), anti-CK10 (Abcam; 1:100), anti-mouse F4/80 (Abcam; 1:100), anti-mouse CD86 (Abcam; 1:100) or anti-mouse CD206 (Abcam; 1:100) antibodies for 8 h at 4 °C. Goat anti-mouse FITC (Abcam;1:200) and goat anti-rabbit TRITC secondary antibodies (Abcam; 1:400) were added for 2 h at room temperature. The fluorescence pictures were collected through a confocal fluorescence microscope (Leica SPII) with 20× magnification.

### Statistical analysis

Data were presented as mean ± standard error of mean (SEM) or standard deviation (SD). Graphpad Prism 7.0 was used to analyze the differences between the experimental groups using *Student t-test*, one way and two-way ANOVA tests followed by a Tukey post hoc test for pairwise comparison. *P* < 0.05 was considered significant.

## Supplementary Information

**Additional file 1: Fig. S1.** Induction of diabetes in C57BL/6J mice by feeding high-fat and high-sugar diet for 4 weeks and subsequent intraperitoneal injections of streptozotocin (STZ) in the last 2 weeks. Quantification of body weight (A) and blood glucose (B) at day 14, day 21 and day 28 upon the treatment of the non-fiber, Un-fiber, RCM-fiber, Un-fiber-BMMSCs and RCM-fiber-BMMSCs in diabetic mice. Differences were analyzed by one-way ANOVA with Tukey's multiple comparison tests. Data were represented as mean ± SD (mice: n ≥ 8) *P < 0.05, **P < 0.01. **Fig. S2.** Anti-CD200R antibody suppressed collagen deposition in the wound regions upon treatment of the RCM-fiber-BMMSCs with anti-CD200R or isotype IgG antibodies. (A). Picrosirius red staining for collagen deposition in the wounds of different groups (scale bar = 50 μm). (B) Quantification of Collagen 1 and Collagen 3 proportions in diabetic wound areas. Data were represented as mean ± SD. Differences were analyzed by one-way ANOVA with Tukey's multiple comparison tests. (Mice: n ≥ 8) *P < 0.05, **P < 0.01. **Fig. S3.** The expression levels of *TNFAIP6* in BMMSCs incubated with the Un-nanofibers, LPS/IFN-γ activated (M1-type) and IL-4 activated (M2-type) RAW264.7 cell membrane coated nanofibers. Data were represented as mean ± SD. Differences were analyzed by one-way ANOVA with Tukey's multiple comparison tests. (Mice: n ≥ 8) *P < 0.05, **P < 0.01. **Table S1.** Primer sequences used in RT-PCR.

## Data Availability

All data are available in the main text or the additional materials.

## References

[1] Service RF (2014). Synthetic biology. Synthetic biologists design ‘living materials’ that build themselves. Science.

[2] Rodrigo-Navarro A, Sankaran S, Dalby MJ, Campo AD, Salmeron-Sanchez M (2021). Engineered living biomaterials. Nat Rev Mater..

[3] He F, Ou Y, Liu J (2022). 3D printed biocatalytic living materials with dual-network reinforced bioinks. Small.

[4] Xin A, Su Y, Feng S (2021). Growing living composites with ordered microstructures and exceptional mechanical properties. Adv Mater.

[5] Zhang D, Zhong D, Ouyang J (2022). Microalgae-based oral microcarriers for gut microbiota homeostasis and intestinal protection in cancer radiotherapy. Nat Commun.

[6] Liu L, Bi M, Wang Y (2021). Artificial intelligence-powered microfluidics for nanomedicine and materials synthesis. Nanoscale.

[7] Winnacker M (2017). Recent advances in the synthesis of functional materials by engineered and recombinant living cells. Soft Matter.

[8] Gartner Z, Hughes A (2019). Getting the measure of living biomaterials. Nature.

[9] Sedel L, Petite H, Bizot P, Nizard R, Meunier A (1999). Biomaterials and the living system. Bull Acad Natl Med..

[10] Gurtner GC, Werner S, Barrandon Y, Longaker MT (2008). Wound repair and regeneration. Nature.

[11] Hunt TK, Burke J, Barbul A, Gimbel ML (1999). Wound healing. Science.

[12] Boulton AJ, Vileikyte L, Ragnarson-Tennvall G, Apelqvist J (2005). The global burden of diabetic foot disease. Lancet.

[13] Shin YC, Lee JH, Jin L (2015). Stimulated myoblast differentiation on graphene oxide-impregnated PLGA-collagen hybrid fibre matrices. J Nanobiotechnology.

[14] Vogel V, Sheetz M (2006). Local force and geometry sensing regulate cell functions. Nat Rev Mol Cell Biol.

[15] Zhang Z, Gupte MJ, Jin X, Ma PX (2015). Injectable peptide decorated functional nanofibrous hollow microspheres to direct stem cell differentiation and tissue regeneration. Adv Funct Mater.

[16] Williams DF (2009). On the nature of biomaterials. Biomaterials..

[17] Tunuguntla RH, Bangar MA, Kim K (2015). Bioelectronic light-gated transistors with biologically tunable performance. Adv Mater.

[18] Gao W, Fang RH, Thamphiwatana S (2015). Modulating antibacterial immunity via bacterial membrane-coated nanoparticles. Nano Lett.

[19] Hu CM, Fang RH, Wang KC (2015). Nanoparticle biointerfacing by platelet membrane cloaking. Nature.

[20] Ding H, Lv Y, Ni D (2015). Erythrocyte membrane-coated NIR-triggered biomimetic nanovectors with programmed delivery for photodynamic therapy of cancer. Nanoscale.

[21] García JR, Quirós M, Han WM (2019). IFN-γ-tethered hydrogels enhance mesenchymal stem cell-based immunomodulation and promote tissue repair. Biomaterials.

[22] Zhang Y, Böse T, Unger RE, Jansen JA, Kirkpatrick CJ, van den Beucken J (2017). Macrophage type modulates osteogenic differentiation of adipose tissue MSCs. Cell Tissue Res.

[23] Zhang Q, Hwang JW, Oh JH (2017). Effects of the fibrous topography-mediated macrophage phenotype transition on the recruitment of mesenchymal stem cells: an in vivo study. Biomaterials.

[24] Yu B, Sondag GR, Malcuit C, Kim MH, Safadi FF (2016). Macrophage-associated osteoactivin/GPNMB mediates mesenchymal stem cell survival, proliferation, and migration via a CD44-dependent mechanism. J Cell Biochem..

[25] Li Y, Zhang D, Xu L (2019). Cell-cell contact with proinflammatory macrophages enhances the immunotherapeutic effect of mesenchymal stem cells in two abortion models. Cell Mol Immunol.

[26] Agrawal CM, Ray RB (2001). Biodegradable polymeric scaffolds for musculoskeletal tissue engineering. J Biomed Mater Res.

[27] Behravesh E, Yasko AW, Engel PS, Mikos AG. Synthetic biodegradable polymers for orthopaedic applications. Clin Orthop Relat Res. 1999. (367 Suppl): S118-29. 10.1097/00003086-199910001-00012 10546641

[28] Athanasiou KA, Niederauer GG, Agrawal CM (1996). Sterilization, toxicity, biocompatibility and clinical applications of polylactic acid/polyglycolic acid copolymers. Biomaterials.

[29] He X, Dong Z, Cao Y (2019). MSC-derived exosome promotes m2 polarization and enhances cutaneous wound healing. Stem Cells Int.

[30] Chen W, Zhang Q, Luk BT (2016). Coating nanofiber scaffolds with beta cell membrane to promote cell proliferation and function. Nanoscale.

[31] Qi Y, Jiang D, Sindrilaru A (2014). TSG-6 released from intradermally injected mesenchymal stem cells accelerates wound healing and reduces tissue fibrosis in murine full-thickness skin wounds. J Invest Dermatol.

[32] Halabian R, Tehrani HA, Jahanian-Najafabadi A, Habibi Roudkenar M (2013). Lipocalin-2-mediated upregulation of various antioxidants and growth factors protects bone marrow-derived mesenchymal stem cells against unfavorable microenvironments. Cell Stress Chaperones.

[33] Bahmani B, Roudkenar MH, Halabian R, Jahanian-Najafabadi A, Amiri F, Jalili MA (2014). Lipocalin 2 decreases senescence of bone marrow-derived mesenchymal stem cells under sub-lethal doses of oxidative stress. Cell Stress Chaperones.

[34] Miao Q, Ku AT, Nishino Y (2014). Tcf3 promotes cell migration and wound repair through regulation of lipocalin 2. Nat Commun.

[35] Whelan DS, Caplice NM, Clover A (2020). Mesenchymal stromal cell derived CCL2 is required for accelerated wound healing. Sci Rep.

[36] Khan B, Rangasamy S, McGuire PG, Howdieshell TR (2013). The role of monocyte subsets in myocutaneous revascularization. J Surg Res.

[37] Stamatovic SM, Keep RF, Mostarica-Stojkovic M, Andjelkovic AV (2006). CCL2 regulates angiogenesis via activation of Ets-1 transcription factor. J Immunol.

[38] Dong S, Zhen F, Xu H, Li Q, Wang J (2021). Leukemia inhibitory factor protects photoreceptor cone cells against oxidative damage through activating JAK/STAT3 signaling. Ann Transl Med.

[39] Negoro S, Kunisada K, Fujio Y (2001). Activation of signal transducer and activator of transcription 3 protects cardiomyocytes from hypoxia/reoxygenation-induced oxidative stress through the upregulation of manganese superoxide dismutase. Circulation.

[40] Xu J, Li Z, Xu P, Yang Z (2012). Protective effects of leukemia inhibitory factor against oxidative stress during high glucose-induced apoptosis in podocytes. Cell Stress Chaperones.

[41] Hu C, Ahmad T, Haider MS (2022). A thermogelling organic-inorganic hybrid hydrogel with excellent printability, shape fidelity and cytocompatibility for 3D bioprinting. Biofabrication.

[42] Yuan Z, Sheng D, Jiang L (2022). Vascular endothelial growth factor-capturing aligned electrospun polycaprolactone/gelatin nanofibers promote patellar ligament regeneration. Acta Biomater.

[43] Chanput W, Mes JJ, Wichers HJ (2014). THP-1 cell line: an in vitro cell model for immune modulation approach. Int Immunopharmacol.

[44] Wang R, Ji Q, Meng C (2020). Role of gingival mesenchymal stem cell exosomes in macrophage polarization under inflammatory conditions. Int Immunopharmacol.

[45] He XT, Li X, Yin Y, Wu RX, Xu XY, Chen FM (2018). The effects of conditioned media generated by polarized macrophages on the cellular behaviours of bone marrow mesenchymal stem cells. J Cell Mol Med.

[46] Wang H, Jiang H, Van De Gucht M, De Ridder M (2019). Hypoxic radioresistance: can ROS be the key to overcome it. Cancers..

[47] Hou J, Han ZP, Jing YY (2013). Autophagy prevents irradiation injury and maintains stemness through decreasing ROS generation in mesenchymal stem cells. Cell Death Dis.

[48] Deng Z, Wang W, Xu X (2021). Biofunction of polydopamine coating in stem cell culture. ACS Appl Mater Interfaces.

[49] van den Bogaard EH, Podolsky MA, Smits JP (2015). Genetic and pharmacological analysis identifies a physiological role for the AHR in epidermal differentiation. J Invest Dermatol.

[50] Priya SG, Jungvid H, Kumar A (2008). Skin tissue engineering for tissue repair and regeneration. Tissue Eng Part B Rev.

[51] Schreurs M, Suttorp CM, Mutsaers H (2020). Tissue engineering strategies combining molecular targets against inflammation and fibrosis, and umbilical cord blood stem cells to improve hampered muscle and skin regeneration following cleft repair. Med Res Rev.

[52] Zhao Y, Su G, Wang Q, Wang R, Zhang M (2021). The CD200/CD200R mechanism in mesenchymal stem cells’ regulation of dendritic cells. Am J Transl Res.

[53] Liu JQ, Hu A, Zhu J, Yu J, Talebian F, Bai XF (2020). CD200-CD200R pathway in the regulation of tumor immune microenvironment and immunotherapy. Adv Exp Med Biol.

[54] Zhang S, Cherwinski H, Sedgwick JD, Phillips JH (2004). Molecular mechanisms of CD200 inhibition of mast cell activation. J Immunol.

[55] Mahmoudian-Sani MR, Rafeei F, Amini R, Saidijam M (2018). The effect of mesenchymal stem cells combined with platelet-rich plasma on skin wound healing. J Cosmet Dermatol.

[56] Sánchez-Sánchez R, Brena-Molina A, Martínez-López V (2015). Generation of two biological wound dressings as a potential delivery system of human adipose-derived mesenchymal stem cells. ASAIO J.

[57] Kucharzewski M, Rojczyk E, Wilemska-Kucharzewska K, Wilk R, Hudecki J, Los MJ (2019). Novel trends in application of stem cells in skin wound healing. Eur J Pharmacol.

[58] Wei Y, Liu Z, Zhu X (2020). Dual directions to address the problem of aseptic loosening via electrospun PLGA @ aspirin nanofiber coatings on titanium. Biomaterials.

[59] Huang Y, Tian C, Li Q, Xu Q (2019). TET1 knockdown inhibits *Porphyromonas gingivalis* LPS/IFN-γ-induced M1 macrophage polarization through the NF-κB pathway in THP-1 cells. Int J Mol Sci..

[60] Li L, Wei C, Cai S, Fang L (2020). TRPM7 modulates macrophage polarization by STAT1/STAT6 pathways in RAW264.7 cells. Biochem Biophys Res Commun.

