# Supplementary material for: Immuno-activated mesenchymal stem cell living electrospun nanofibers for promoting diabetic wound repair
Source: J Nanobiotechnology. 2022 Jun 21;20:294. doi: 10.1186/s12951-022-01503-9 (PMC9210587; doi:10.1186/s12951-022-01503-9)
Supplement: Supplementary file 1 — Additional file 1: Fig. S1. Induction of diabetes in C57BL/6J mice by feeding high-fat and high-sugar diet for 4 weeks and subsequent intraperitoneal injections of streptozotocin (STZ) in the last 2 weeks. Quantification of body weight (A) and blood glucose (B) at day 14, day 21 and day 28 upon the treatment of the non-fiber, Un-fiber, RCM-fiber, Un-fiber-BMMSCs and RCM-fiber-BMMSCs in diabetic mice. Differences were analyzed by one-way ANOVA with Tukey's multiple comparison tests. Data were represented as mean ± SD (mice: n ≥ 8) *P < 0.05, **P < 0.01. Fig. S2. Anti-CD200R antibody suppressed collagen deposition in the wound regions upon treatment of the RCM-fiber-BMMSCs with anti-CD200R or isotype IgG antibodies. (A). Picrosirius red staining for collagen deposition in the wounds of different groups (scale bar = 50 μm). (B) Quantification of Collagen 1 and Collagen 3 proportions in diabetic wound areas. Data were represented as mean ± SD. Differences were analyzed by one-way ANOVA with Tukey's multiple comparison tests. (Mice: n ≥ 8) *P < 0.05, **P < 0.01. Fig. S3. The expression levels of TNFAIP6 in BMMSCs incubated with the Un-nanofibers, LPS/IFN-γ activated (M1-type) and IL-4 activated (M2-type) RAW264.7 cell membrane coated nanofibers. Data were represented as mean ± SD. Differences were analyzed by one-way ANOVA with Tukey's multiple comparison tests. (Mice: n ≥ 8) *P < 0.05, **P < 0.01. Table S1. Primer sequences used in RT-PCR. [file 12951_2022_1503_MOESM1_ESM.docx]

**Supplementary Materials**

**Immuno-activated mescenchymal stem cell living electrospun nanofibers for promoting diabetic wound repair**

Shaoying Gao^a,b^ , Tao Chen^a^, Zhen Wang^c^, Ping Ji^b^, Lin Xu^d*^, Wenguo Cui^c*^, Ying Wang^b*^

**
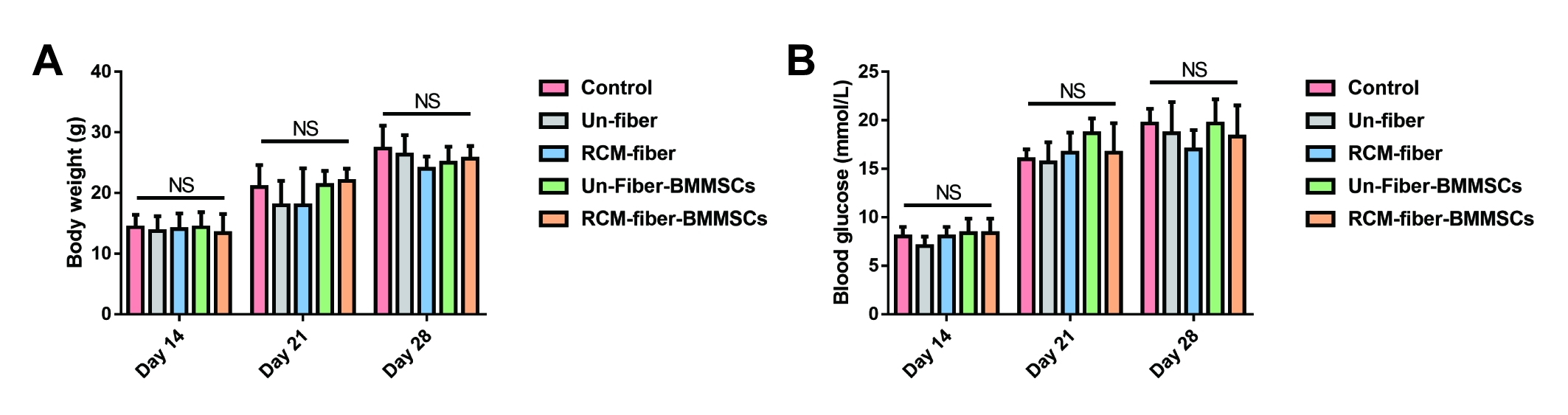
**

**Figure S1**. Induction of diabetes in C57BL/6J mice by feeding high-fat and high-sugar diet for 4 weeks and subsequent intraperitoneal injections of streptozotocin (STZ) in the last 2 weeks. Quantification of body weight (A) and blood glucose (B) at day 14, day 21 and day 28 upon the treatment of the non-fiber, Un-fiber, RCM-fiber, Un-fiber-BMMSCs and RCM-fiber-BMMSCs in diabetic mice. Differences were analyzed by one-way ANOVA with Tukey's multiple comparison tests. Data were represented as mean ± SD (n ≥ 8) **P* < 0.05, ***P* < 0.01


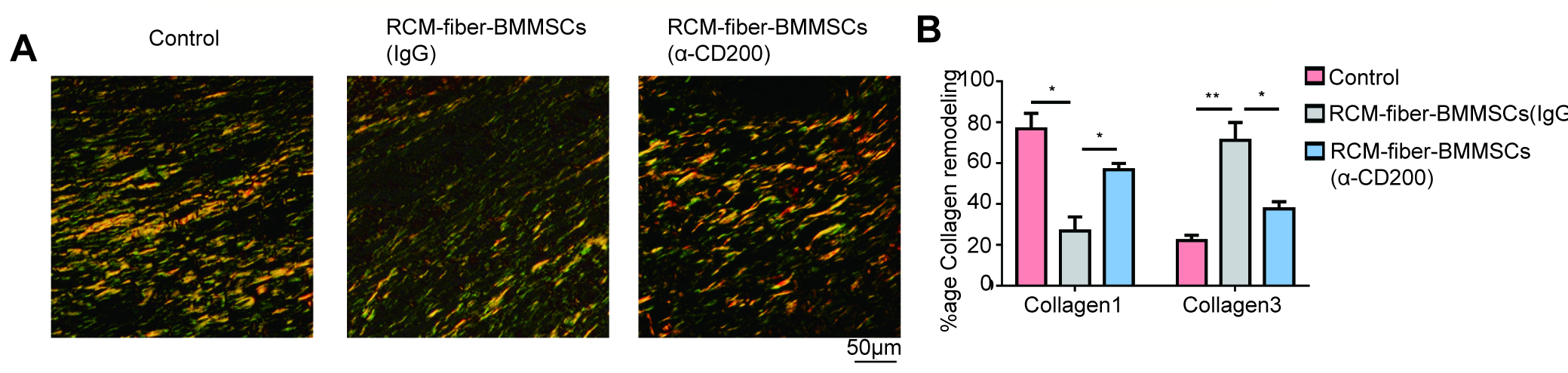


**Figure S2.** Anti-CD200R antibody suppressed collagen deposition in the wound regions upon treatment of the RCM-fiber-BMMSCs with anti-CD200R or isotype IgG antibodies. **(**A). Picrosirius red staining for collagen deposition in the wounds of different groups(scale bar = 50μm). (B) Quantification of collagen 1 and collagen 3 proportions in diabetic wound areas. Data were represented as mean ± SD. Differences were analyzed by one-way ANOVA with Tukey's multiple comparison tests. (n ≥ 8) **P* < 0.05, ***P* < 0.01


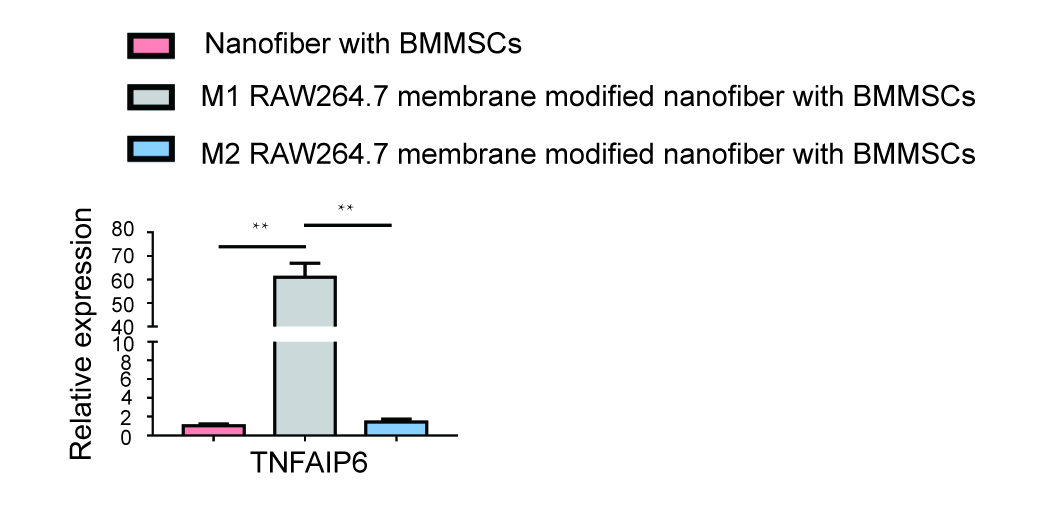


**Figure S3** The expression levels of *TNFAIP6* in BMMSCs incubated with the Un-nanofibers, LPS/IFN-γ activated (M1-type) and IL-4 activated (M2-type) RAW264.7 cell membrane coated nanofibers. Data were represented as mean ± SD. Differences were analyzed by one-way ANOVA with Tukey's multiple comparison tests. (n ≥ 8) **P* < 0.05, ***P* < 0.01

| Primer name | Sequence (5′-3′) |
| --- | --- |
| GAPDH | TGTTGAAGTCGCAGGAGACAACCT  AACCTGCCAAGTATGATGACATCA |
| TNFAIP6 | GGCTGGCAGATACAAGCTCA  TCAAATTCACATACGGCCTTGG |
| CD200 | GCCTTACCCTCTATGTACAGCC  TCCCAGTACCCTTCCAGGAG |
| Lif | GCTCTCCTGTGCTGCTCTTAC  GGAGGGTAAATCTAGGCGACA |
| Lcn2 | GGCCAGTTCACTCTGGGAAA  TGGCGAACTGGTTGTAGTCC |
| CcL2 | CCACAACCACCTCAAGCACT  AGGCATCACAGTCCGAGTCA |
| IL-10 | TTTTCACAGGGGAGAAATCG  CCAAGCCTTATCGGAAATGA |
| IL-1β | GGTGTGTGACGTTCCCATTA  ATTGAGGTGGAGAGCTTTCAG |

**Table S1** Murine and human primer sequences used in RT-PCR. Target genes needed to be RT-PCR(left). Primer sequences of target genes were applied in RT-PCR(right).
